# Combined activation of artificial and natural ion channels for disrupting mitochondrial ion homeostasis towards effective postoperative tumor recurrence and metastasis suppression

**DOI:** 10.7150/thno.94855

**Published:** 2024-05-27

**Authors:** Panqin Ma, Zheng Luo, Qi Wang, Ying Chen, Feng Liu, Changliang Ren, Caisheng Wu, Zibiao Li, Yun-Long Wu

**Affiliations:** 1 Fujian Provincial Key Laboratory of Innovative Drug Target Research and State Key Laboratory of Cellular Stress Biology, School of Pharmaceutical Sciences, Xiamen University, Xiamen 361102, China.; 2 Institute of Materials Research and Engineering, A*STAR (Agency for Science, Technology and Research), 2 Fusionopolis Way, Innovis, #08-03, Singapore 138634, Singapore.; 3 Institute of Sustainability for Chemicals, Energy and Environment (ISCE2), A*STAR (Agency for Science, Technology and Research), 1 Pesek Road, Jurong Island, Singapore, 627833, Singapore.; 4 Shenzhen Research Institute of Xiamen University, Shenzhen, Guangdong 518057, China.

**Keywords:** mitochondrial ion channels, artificial K^+^ channels, synergistic effect, mitochondrial homeostasis, tumor recurrence and metastasis

## Abstract

**Rationale**: Pharmacological targeting of mitochondrial ion channels is developing as a new direction in cancer therapy. The opening or closing of these channels can impact mitochondrial function and structure by interfering with intracellular ion homeostasis, thereby regulating cell fate. Nevertheless, their abnormal expression or regulation poses challenges in eliminating cancer cells, and further contributes to metastasis, recurrence, and drug resistance.

**Methods**: We developed an engineered mitochondrial targeted delivery system with self-reinforcing potassium ion (K^+^) influx via amphiphilic mitochondrial targeting polymer (TMP) as carriers to co-deliver natural K^+^ channel agonists (Dinitrogen oxide, DZX) and artificial K^+^ channel molecules (5F8).

**Results**: Using this method, DZX specifically activated natural K^+^ channels, whereas 5F8 assembled artificial K^+^ channels on the mitochondrial membrane, leading to mitochondrial K^+^ influx, as well as oxidative stress and activation of the mitochondrial apoptotic pathway.

**Conclusion**: The synergistic effect of 5F8 and DZX presents greater effectiveness in killing cancer cells than DZX alone, and effectively inhibited tumor recurrence and lung metastasis following surgical resection of breast cancer tumors in animal models. This strategy innovatively integrates antihypertensive drugs with artificial ion channel molecules for the first time to effectively inhibit tumor recurrence and metastasis by disrupting intracellular ion homeostasis, which will provide a novel perspective for postoperative tumor therapy.

## Introduction

Ion channels or ion transporters are specialized pore-forming membrane proteins that can only allow charged ions of a certain size or charge to pass. They widely appear on the membranes of various cells or organelles, and are involved in the regulation of virtually all basic cellular processes including the malignant phenotype of cancer cells 1, 2. Among the ion channels, potassium ion (K^+^) channels are the most diverse in the plasma membrane ion channel family 3, which play a key role in the processes of cell proliferation, differentiation, invasion, energy metabolism and apoptosis, etc. Potassium channels exhibit the highest variability and the most frequently altered expression in many tumor types. Plenty of studies have shown that the regulation of potassium channel activity can effectively affect the apoptosis, metastasis, and drug resistance of cancer cells 4-7. In recent years, mitochondrial potassium channels are emerging as new pharmacological targets for cancer treatment 8, 9. Regulating the opening or closing of mitochondrial potassium channels disrupts the ion balance of mitochondria, thereby affecting the energy conversion process, mitochondrial structure and function, and the production of reactive oxygen species (ROS), and thus the fate of cancer cells 10, 11. For example, regulation of the activity of the mitochondrial K^+^ channel Kv1.3 12 or Kv1.5 13 in cancer cells has been shown to be effective in tumor suppression. However, in view of the differences in the expression or regulation of potassium ion channels in different cancer cells, effectively regulating mitochondrial potassium ion channels still faces huge challenges.

Dinitrogen oxide (DZX), a mitochondrial potassium channel activator, specifically regulates the opening of potassium channels to change the intracellular potassium concentration and inhibits succinate dehydrogenase (SDH) in the mitochondrial respiratory chain, increasing mitochondrial permeability. It is commonly used clinically to rapidly lower blood pressure and treat persistent hyperinsulinemic hypoglycemia 14, 15. Recently, studies have shown that it also shows potential against tumors. It shows a concentration-dependent inhibitory effect on a variety of tumor cells 16-19 such as human hepatoma cells (HepG2), human colon cancer cells (HT29 cells), human lung cancer cells (H1299 cells), which may be attributed to the fact that it can induce mitochondrial depolarization by regulating the flow of K^+^, thereby damaging the mitochondrial structure and function, causing upregulation of ROS and reduction of ATP 20-23. However, the low expression or inhibitory regulation of K^+^ channels in cancer cell mitochondria makes them difficult to effectively eliminate 13, 24, 25. Fortunately, thanks to the development of artificial ion channel technology, the above problems may be effectively solved. Artificial ion channels are mimics of natural channel proteins that can also transmit certain ions highly selectively 26-29. Artificial potassium channels have shown promising tumor suppression effects 30-34. They promote the flow of K^+^ from high concentration to low concentration by creating potassium channels on the membrane, which disrupt intracellular K^+^ homeostasis and induce oxidative stress, thereby destroying the membrane structure and leading to apoptosis of cancer cells. It would be ideal if the artificial ion channel could be identified to improve the ability of natural potassium channel to transport K^+^, creating a synergistic therapy of “1+1>2” to achieve the desired antitumor effect. This cancer treatment strategy, which combines both artificial and natural ion channels to disrupt ion homeostasis, will provide a new insight into a wide range of current cancer treatments 35-41 and may lead to unexpected cancer treatment effects.

**Figure 1:**
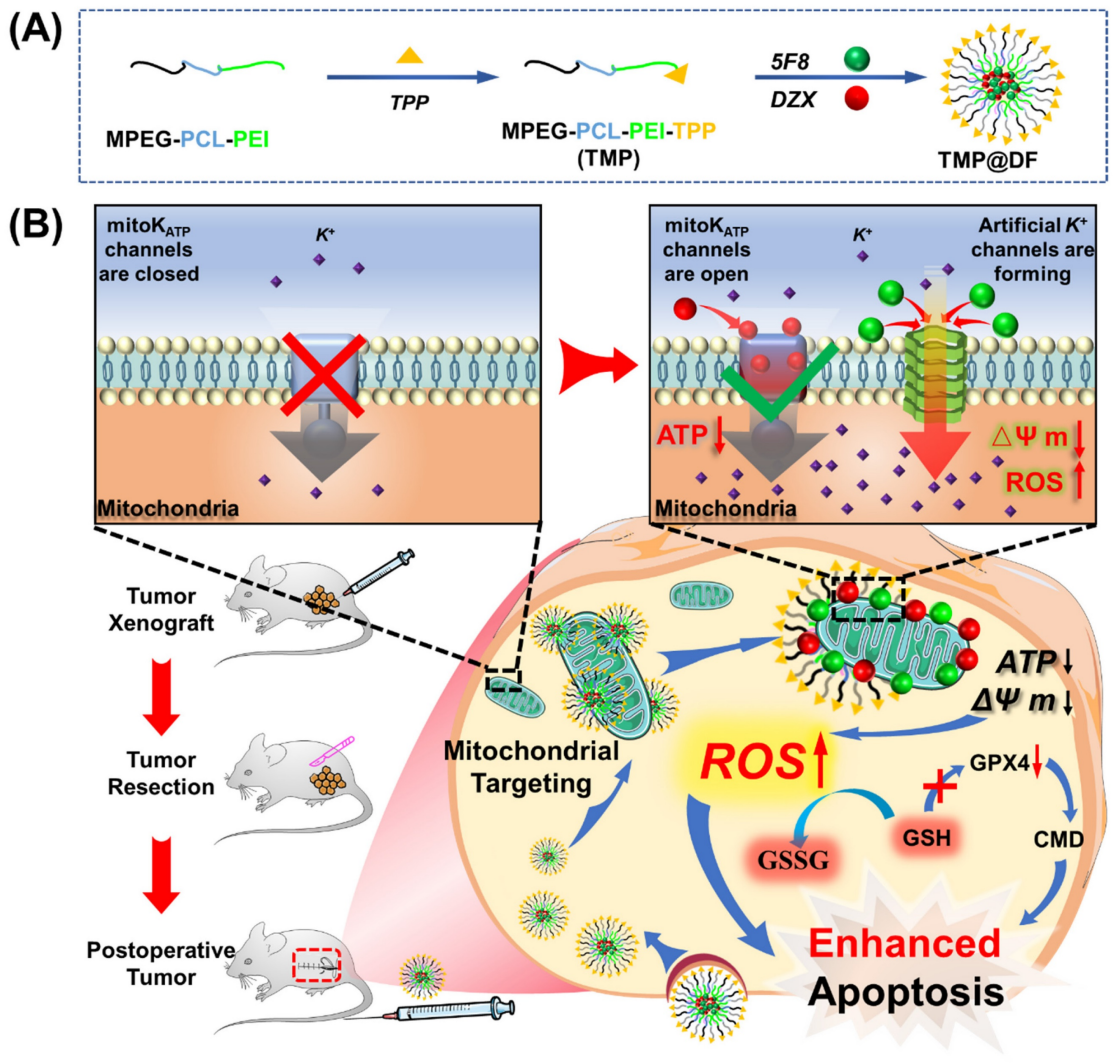
Schematic diagram of codelivery artificial K^+^-selective ion channel and diazoxide on disruption K^+^ homeostasis of mitochondria in postsurgical cancer recurrence and metastasis strategy. (A) The synthesis process of TMP@DF. (B) The mechanism of TMP@DF at the mitochondrial membrane of the tumor site. This strategy interferes with mitochondrial K^+^ homeostasis by causing intracellular potassium influx into mitochondria, thereby disrupting mitochondrial structure and function, such as restricting ATP synthesis, decreasing mitochondrial membrane potential, upregulating ROS, and consuming GSH, inhibiting GPX4 protein expression, damaging the structure of the cellular membrane, and then activating mitochondrial apoptotic pathways to induce apoptosis in postoperative tumor recurrence and metastasis.

In the present work, we developed an engineered mitochondrial targeted delivery system (**Figure 1**) with self-enhancing K^+^ influx function via using amphiphilic mitochondrial targeting polymer (MPEG-PCL-PEI-TPP, TMP) as a carrier to co-deliver natural K^+^ channel agonists (DZX) and artificial K^+^ channel molecules (5F8) 31 synthesized by our research group. It can selectively deliver drug molecules to cancer cell mitochondria, utilizing DZX to specifically activate natural potassium ion channels, and 5F8 assembles artificial K^+^-selective ion channels on the mitochondrial membrane, thereby causing K^+^ inflow to destroy intracellular ions balance. It would lead to mitochondrial oxidative stress and inhibition of glutathione peroxidase (GPX4) activity, thereby causing accumulation of ROS, damaging mitochondrial structure and function, including a decrease in mitochondrial membrane potential, a decrease in ATP synthesis, and an increase in the permeability of mitochondrial membranes, thereby activating the mitochondrial apoptosis pathway, manifested by an increase in activities of Caspase-3 and Caspase-9. Interestingly, the synergistic effects of 5F8 and DZX was 60 times greater in inhibiting cancer cell proliferation than DZX targeting mitochondria alone, and significantly inhibited tumor recurrence and lung metastasis after surgical removal of breast cancer tumors in a mouse subcutaneous tumor model. This work will provide a pioneering perspective on the motorized joint action of artificial ion channels and natural ion channels, and provide an alternative therapeutic option for recurrence and metastasis of surgically resected tumors in clinical practice.

## Results and Discussion

### Fabrication and characterization of polymeric nanoparticles (TMP@DF) and its mitochondrial targetability verification

**Figure 2:**
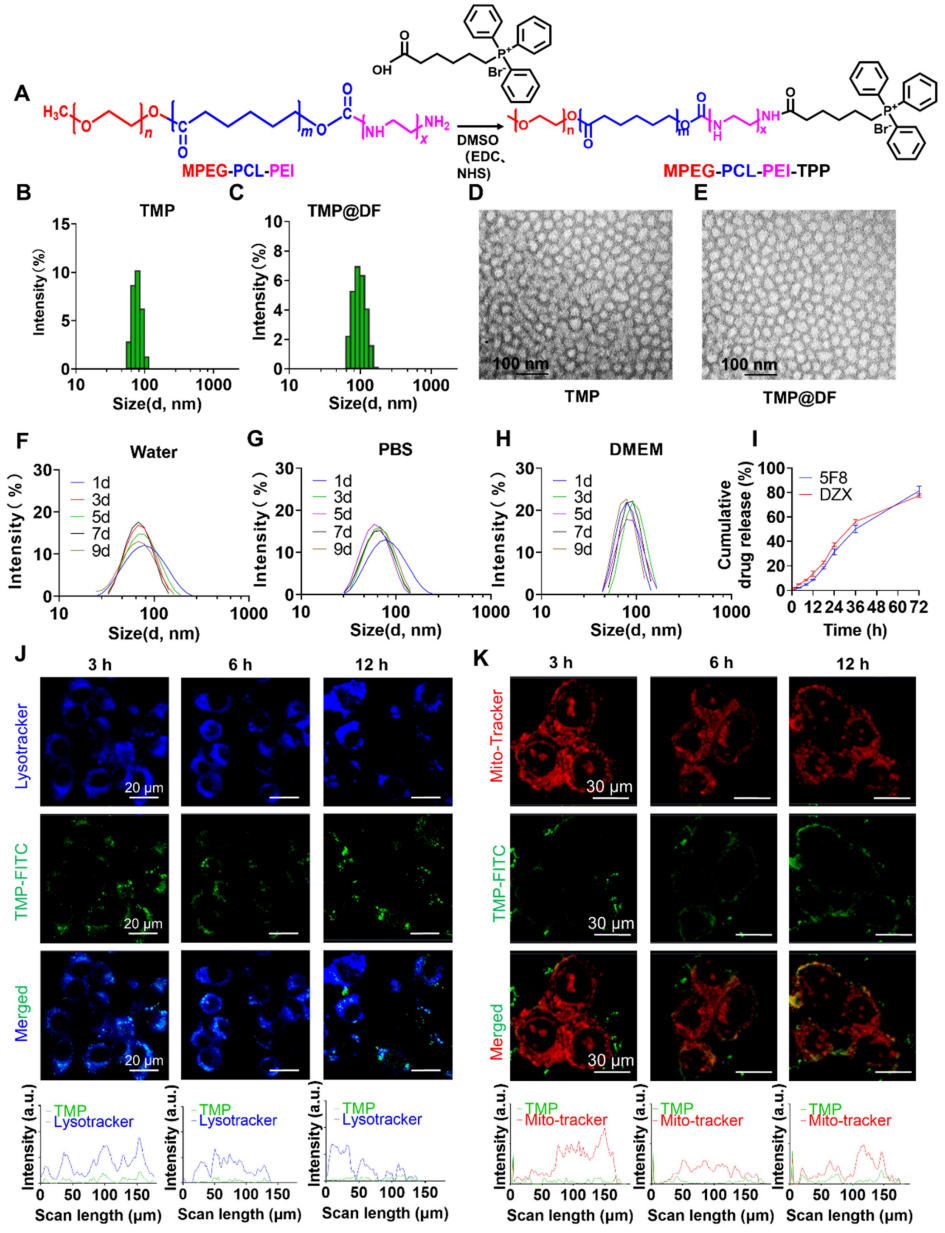
(A) Synthetic route of TMP copolymer. Dynamic particle size-distribution graphs of (B) TMP and (C) TMP@DF. TEM images of the particles of (D) TMP and (E) TMP@DF. Stability of particle size of micelles in different solvents, including (F) water, (G) PBS and (H) DMEM media. (I) Cumulative drug release curve of TMP@DF NPs in pH 7.4 phosphate buffer solution. (J) Lysosomal colocalization of 4T1 cells at different times after treatment, and lysosomal colocalization analysis of 4T1 cells (blue represents lysosomes, green represents TMP polymer). (K) Mitochondrial colocalization imaging of 4T1 cells treated with TMP for 12 h.

The synthesis of MPEG-PCL-PEI (MPP) copolymer was reported previously 42, 43. A novel amphiphilic mitochondria-targeting copolymer MPEG-PCL-PEI-TPP (TMP) was designed and successfully synthesized according to the synthetic route shown in **Figure 2A**, where TPP is the mitochondria-targeting component. Specifically, TMP was obtained by amidation reaction between MPP and TPP, and the emerging signal in its ^31^P NMR (**Figure S1**) at 121.6 ppm was a phosphorus element in TPP, indicating the presence of a TPP fragment component in TMP. The results confirm the successful synthesis of TMP.

In aqueous solution, the obtained copolymer can self-assemble into micelles consisting of MPEG and PEI hydrophilic shells and PCL hydrophobic nuclei. The TPP portion of the surface confers the mitochondrial targeting properties of the micelles, while the hydrophobic core located inside can be loaded with the lipid-soluble artificial K^+^ channel molecules (5F8) and diazoxide (DZX), which significantly improves the water solubility of 5F8 and DZX. The results of DLS showed that the average particle sizes of TMP and TMP@DF group were 78.8 nm and 91.3 nm, respectively (**Figure 2B-C**). The morphologies of TMP and TMP@DF were evaluated by TEM, and both were spherical nanoparticles with particle size less than 100 nm (**Figure 2D-E**). We further evaluated the stability of TMP@DF NPs in water (**Figure 2F**), PBS buffer at pH7.4 (**Figure 2G**) and DMEM (**Figure 2H**) at different times. The results showed that TMP@DF maintained good stability in water, PBS and DMEM solution until the day 9. These results indicated that both 5F8 and DZX can be effectively encapsulated by TMP through hydrophobic interaction, and TMP@DF NPs had good structural stability. The drug loading content of 5F8 and DZX in the nanoparticles was about 7.18% and 7.09%, and the entrapment efficiency was about 78.67% and 77.64%, respectively.

Next, to fully validate the potential of TMP@DF as a good drug delivery platform, we further evaluated the release ability *in vitro*, cellular uptake ability and mitochondria-targeting performance. First, the cumulative release of 5F8 in pH 7.4 phosphate buffer was measured by HPLC. The results showed that TMP@DF NPs could achieve a slow release of 5F8 and DZX for 72 hours with a maximum cumulative release of close to 80% (**Figure 2I**). Considering that most micelles must escape from lysosomes to reach the target site, the lysosomal escape ability of micelles in cells was also evaluated. As shown in **Figure 2J**, the results showed that the complex micelles showed lysosome escape trend after 6 h, and complete lysosome escape was achieved after 12 h (Pearson coefficients ~0.16281). More importantly, as shown in **Figure 2K**, the green fluorescent signal of TMP maintained good accumulation in the mitochondrial region (red fluorescent), indicating that the presence of TPP conferred mitochondrial targeting activity to TMP@DF copolymer micelles (Pearson coefficients ~0.71151). These results suggest that TMP@DF NPs have a great potential to co-deliver both 5F8 and DZX into the mitochondria of cancer cells to construct K^+^-selective ion channels, while activating endogenous ATP-sensitive potassium channels (mitoK_ATP_).

### 5F8/DZX synergistically inhibiting cell viability* in vitro*

To evaluate the toxicity of 4T1 tumor cells after treatment with mitochondria-targeted codelivery both 5F8 and DZX, we compared the antitumor effects of different drug combinations. Firstly, the proliferation inhibition ability of free DZX in 4T1 cells (**Figure S2**(a)), TMP@5F8, TMP@DZX, and TMP@DF to treat 4T1 cells (Figure S2(b)) were investigated. The results showed that the inhibition of 4T1 cells by DZX was weak with an IC_50_ of 797.4 μM. While the proliferation of 4T1 tumor cells was significantly inhibited in the mitochondrial targeting group, more crucially, the IC_50_ for 4T1 tumor cell proliferation in group TMP@DF NPs (1.38 μM) was more than 60-fold higher than that in group TMP@DZX (88.27 μM). This result suggests that the combination of 5F8 and DZX may have a significantly synergistic effect for therapeutics in breast cancer cells. In spite of this development of results, we venture to hypothesize that 5F8 might accelerate the ability of mitoK_ATP_ to transport potassium ions, thereby causing significant cytotoxicity.

**Figure 3:**
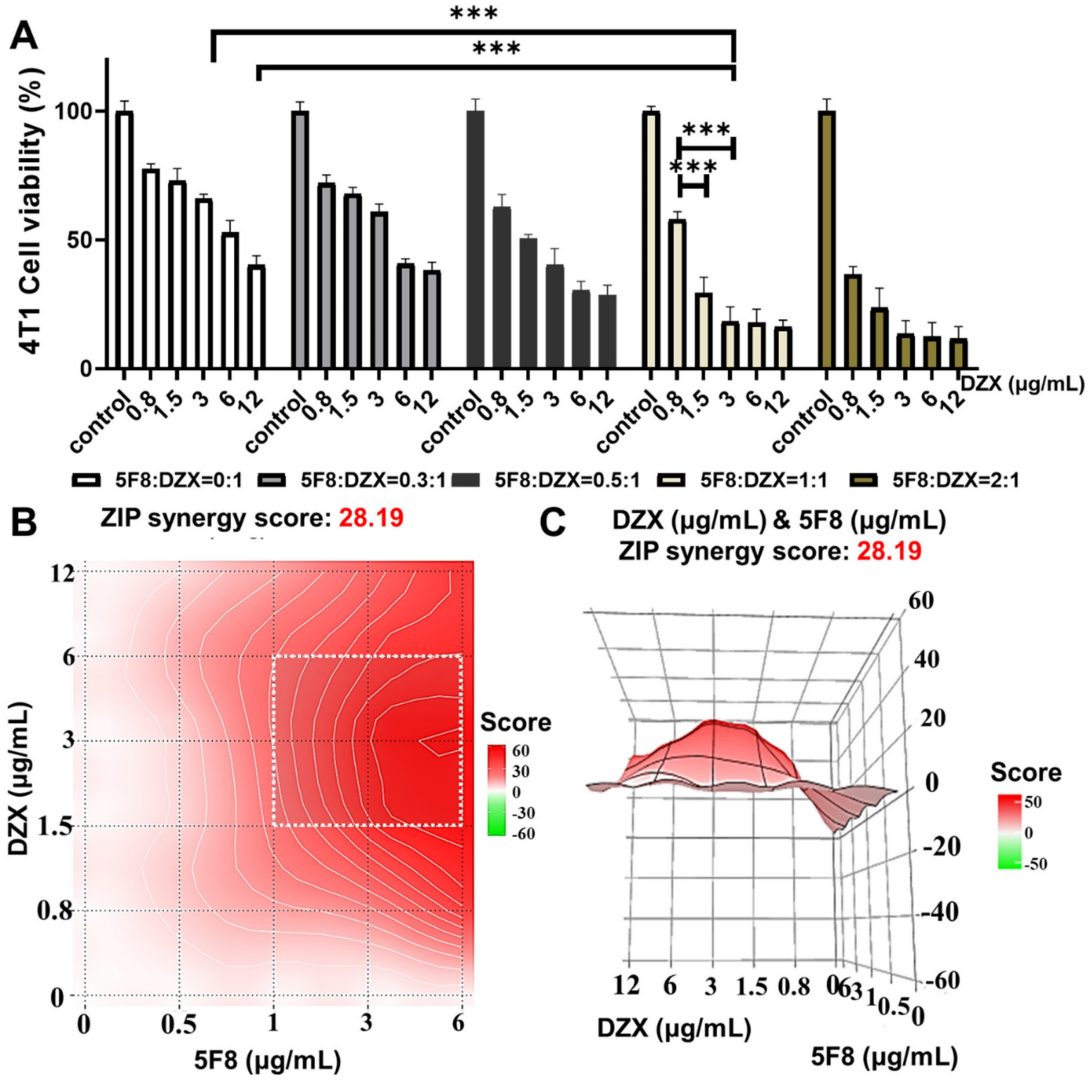
Combining 5F8 with DZX has a synergistic effect on 4T1 cells. (A) 4T1 cells were treated with different concentrations of 5F8 and DZX alone or in combination. The graph of synergy 2D (B) and 3D (C) plots showing drug synergism of 4T1 cells, after exposure to fixed dose ratios of 5F8 and DZX with 3 μg/mL and more of 1.5 μg/mL, respectively. The combined treatment was co-administered at the same time. Synergy score: < -10 (antagonism, green), -10 to 10 (additivity, white) and > 10 (synergism, red). Data are presented as means ± SD from three independent experiments. Statistical comparisons were analyzed by One-way ANOVA Tukey's multiple comparisons test. ^*^p < 0.05, ^**^p < 0.01, ^***^p < 0.001, n.s., not significant.

To further verify the mitochondria-targeting synergistic effect of 5F8 and DZX, we prepared TMP@DF nanoparticles loaded with the mass concentration ratios of 5F8 and DZX as 0:1, 0.3:1, 0.5:1, 1:1 and 2:1, respectively. The above nanoparticles were screened for specific therapeutic effects on 4T1 cells. The results showed (**Figure 3A**) that the TMP@DF NPs with 5F8 and DZX ratio of 1:1 were significantly increased in anticancer effect compared to the TMP@DZX control group with any concentration of DZX (*p* < 0.001). More interestingly, for 293T cells, this mitochondrial-targeting combination of 5F8 and DZX had no significant inhibitory effect on cell viability compared to untreated controls (**Figure S3**). This result suggested the mitochondria-targeted polymeric NPs had almost no effect on normal cells. In addition, we evaluated the drug interactions using Synergy Finder web application (**Figure 3B-C**). For the application, the synergy score for a drug combination was averaged over all the dose combination measurements to give a positive (synergy, red) and negative (antagonism, green) synergy score values. The synergy score calculated from these data was 28.19, pointing to a synergistic action of DZX and 5F8 in reducing cell viability. To further scrutinise the overall synergy between 5F8 and DZX via mitochondrial targeting administration, the detail value of cell viability was evaluated. As presented in **Figure S4**, regardless of the concentration up to 12 μg/mL, the proliferation of 4T1 tumor cells could not be effectively inhibited after treatment with DZX alone. As the concentration of 5F8 and DZX increased to 1.5 μg/mL, it was apparent the antitumor synergistic effect was remarkably enhanced (active suppression rate reached more than 80%). We further validated the excellent anti-tumor effect of TMP@DF using a live/dead staining assay kit (**Figure S5**). Thus, mitochondria-targeted co-delivery of 5F8 and DZX could exert a strong synergetic antitumor effect defined by “1+1>2”, and furthermore might suppress the tumor recurrence and metastasis.

### Synergetic antitumor mechanism and efficacy of TMP@DF NPs on mitochondrial function and structure

**Figure 4:**
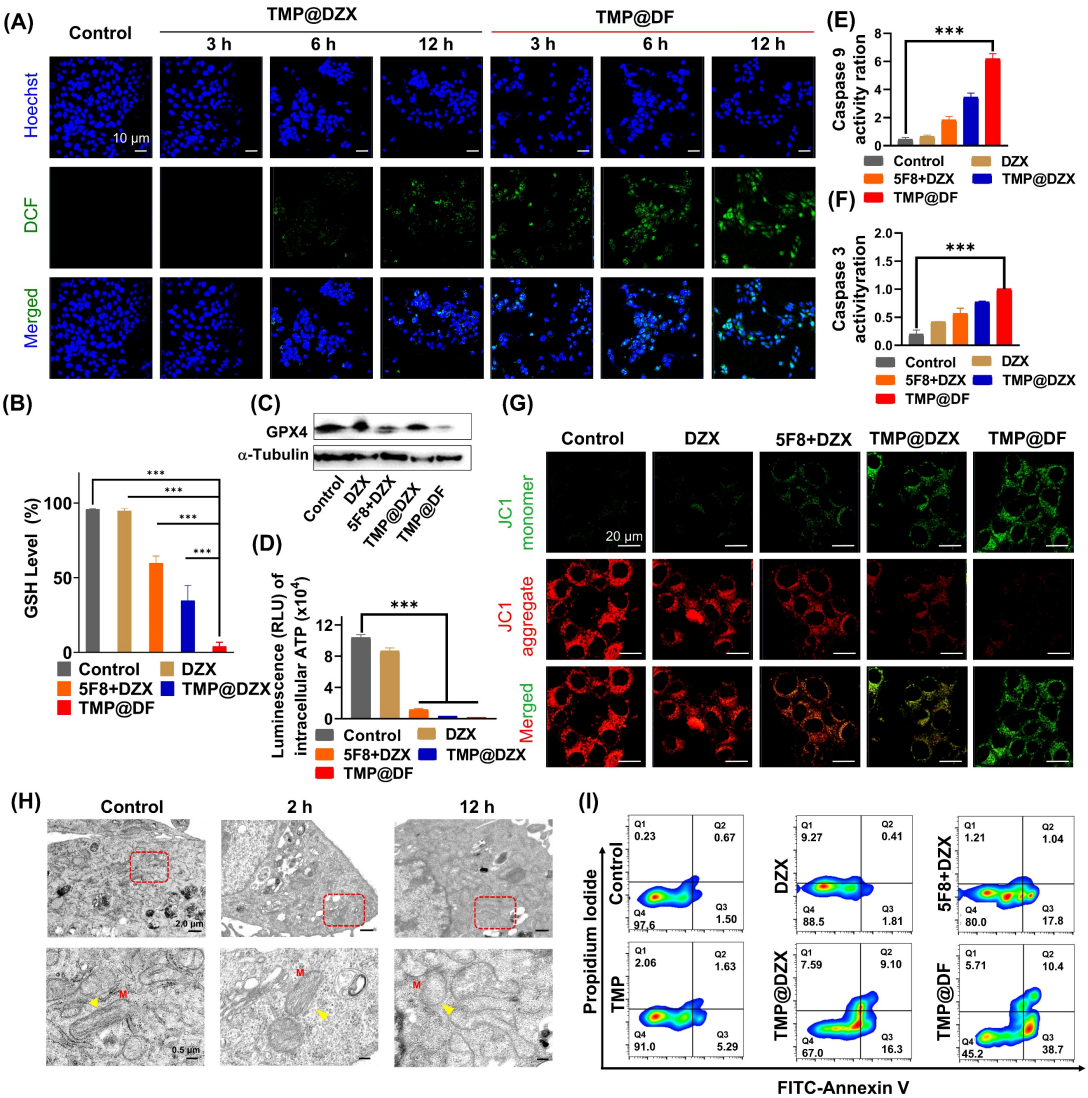
*In vitro* study of the molecular mechanism of the antitumor effect of TMP@DF. (A) Confocal fluorescence images of ROS generation in 4T1 cells treated with TMP@DZX and TMP@DF NPs at different times measured by DCF. (B) Intracellular GSH level of 4T1 cells after treated with different treatments. n = 6, ^***^p < 0.001. (C) Western blot analysis of GPX 4 expression in 4T1 cells. (D) ATP levels in 4T1 cells after different sample treatments. Activities of Caspase-9 (E) and Caspase-3 (F) determined by microplate reader in 4T1 cells after different sample treatments. (G) Confocal fluorescence images of mitochondrial membrane potential analysis of 4T1 cells treated with different sample treatments. (H) TEM images of mitochondria ultrastructure. M: Mitotracker. (I) Cell apoptotic rate detected by flow cytometry. Data are presented as means ± SD from three independent experiments. Statistical comparisons were analyzed by One-way ANOVA Tukey's multiple comparisons test. ^*^p < 0.05, ^**^p < 0.01, ^***^p < 0.001, ^****^p < 0.0001, n.s., not significant.

Given the superior cellular uptake ability and precise mitochondrial targeting properties of TMP@DF NPs, we evaluated the antitumor therapeutic effect and its effect on mitochondrial function and structure *in vitro*. First, we assessed the ability of NPs (TMP@DZX and TMP@DF) to generate intracellular ROS in different times. As shown in **Figure 4A**, the TMP@DZX NPs group showed little green fluorescence (DCFH-DA) until 6 hours. Notably, TMP@DF NPs had more green fluorescence after treatment 4T1 cells with 3 hours than TMP@DZX group at 12 hours, moreover the fluorescence intensity was stronger at 12 hours by treatment with TMP@DF group, which suggesting that intracellular ROS efficiently was generated due to the synergetic mitochondrial-targeted co-delivery 5F8 and DZX. To further verify causes of mitochondrial damage, we further evaluated the effect of TMP@DF NPs on mitochondrial function and architecture. As shown in **Figure 4G**, the results showed that TMP@DF could significantly reduce the membrane potential of mitochondria (Δφ m), manifested by a gradual increase in green fluorescence, indicating the functional damage of mitochondria. It can be tentatively hypothesized that the intracellular ROS are caused by mitochondrial damage. Furthermore, glutathione peroxidase 4 (GPX4), a lipid repair enzyme, acts as an important antioxidant of cell membranes by regulating glutathione (GSH) production to remove excess ROS in cells. As seen in** Figure 4B**, the GSH level significantly declined after the treatment of TMP@DF NPs, which in turn was accompanied by conversion to oxidized glutathione (GSSG). In addition, the content of GPX4 protein was measured by western blot (**Figure 4C** and** S6a**). As expected, the levels of GPX4 declined to some extent among TMP@DF NPs, which was also consistent with results of GSH levels. Furthermore, change in ATP synthesis is another important characteristic of mitochondria. As shown in **Figure 4D**, the ATP synthesis in mitochondria was significantly reduced after mitochondria-targeted DZX administration alone compared to free DZX, while ATP synthesis was almost undetectable when mitochondria were co-delivered with 5F8 and DZX. This suggests that mitochondria-targeted administration of 5F8 and DZX effectively inhibits the ATP synthesis in mitochondria through synergistic effects, which subsequently affects the energy supply function of mitochondria and is a major factor in apoptosis.

In order to fully understand the changes of mitochondrial ultrastructure after treatment with TMP@DF NPs, 4T1 tumor cells were fixed in 2.5% glutaraldehyde and collected for TEM observation (**Figure 4H**). For control, the mitochondria remained normal with clear internal architecture, while mitochondria swelling, damage of the inner ridge, membrane structure blurring and degeneration were observed in cells after treatment with TMP@DF NPs in 12 h (indicated by yellow triangles), illustrating that apoptosis occurred in 4T1 tumor cells. In addition, caspase 9- and caspase 3-dependent apoptosis pathways were activated by this treatment (**Figure 4E-F**), especially in the TMP@DF NPs group, indicating that this synergistical interference of mitochondrial K^+^ homeostasis stratagem could activate the caspase-dependent apoptosis paths for tumor therapy. The mechanistic results in research aforementioned will help induce the apoptosis of postoperative cancer cells, using Annexin V/PI kit to validation the programmed tumor cell death induced by TMP@DF NPs. As shown in **Figure 4I and S6b**, more apoptotic cells appeared in the TMP@DF NPs group than in the free DZX group. Notably, 5F8 and DZX, as well as mitochondria-targeted DZX administration alone also stimulated apoptosis to some extent compared to free DZX. However, the stimulation of apoptosis in tumor cells was more remarkable after mitochondrial co-delivery of 5F8 and DZX, and the effect of TMP on apoptosis was minimal, suggesting that in the TMP@DF NPs group, apoptosis was mediated by the release of 5F8 and DZX from NPs. In addition, we also explored the influence of TMP@DF on the phenotype of macrophages, as shown in **Figure S7**. With the increase of the concentration of TMP@DF nanoparticles, the number of anti-tumor type M1 macrophages also increases, while it has little effect on the immune-suppressed M2 macrophages, which indicates that it can effectively increase the number of M1 macrophages in the tumor microenvironment, thereby better killing tumor cells and inhibiting their metastasis. Therefore, we believe that TMP@DF NPs have potential as tumor therapeutic agents. This further substantiates that TMP@DF causes mitochondrial structural damage by reducing mitochondrial membrane potential and up-regulating ROS levels, which will consume endogenous GSH, prevent the express of GPX4 protein, limit the synthesis of ATP, and activate the paths of caspase-dependent apoptosis, thus significantly promoting tumor cell apoptosis and improving anti-tumor efficiency. We posit that the strategy of achieving antitumor effects based on interfering mitochondrial K^+^ homeostasis will provide the possibility to overcome tumor recurrence and metastasis.

### Effect of TMP@DF on K^+^ homeostasis in mitochondria

**Figure 5:**
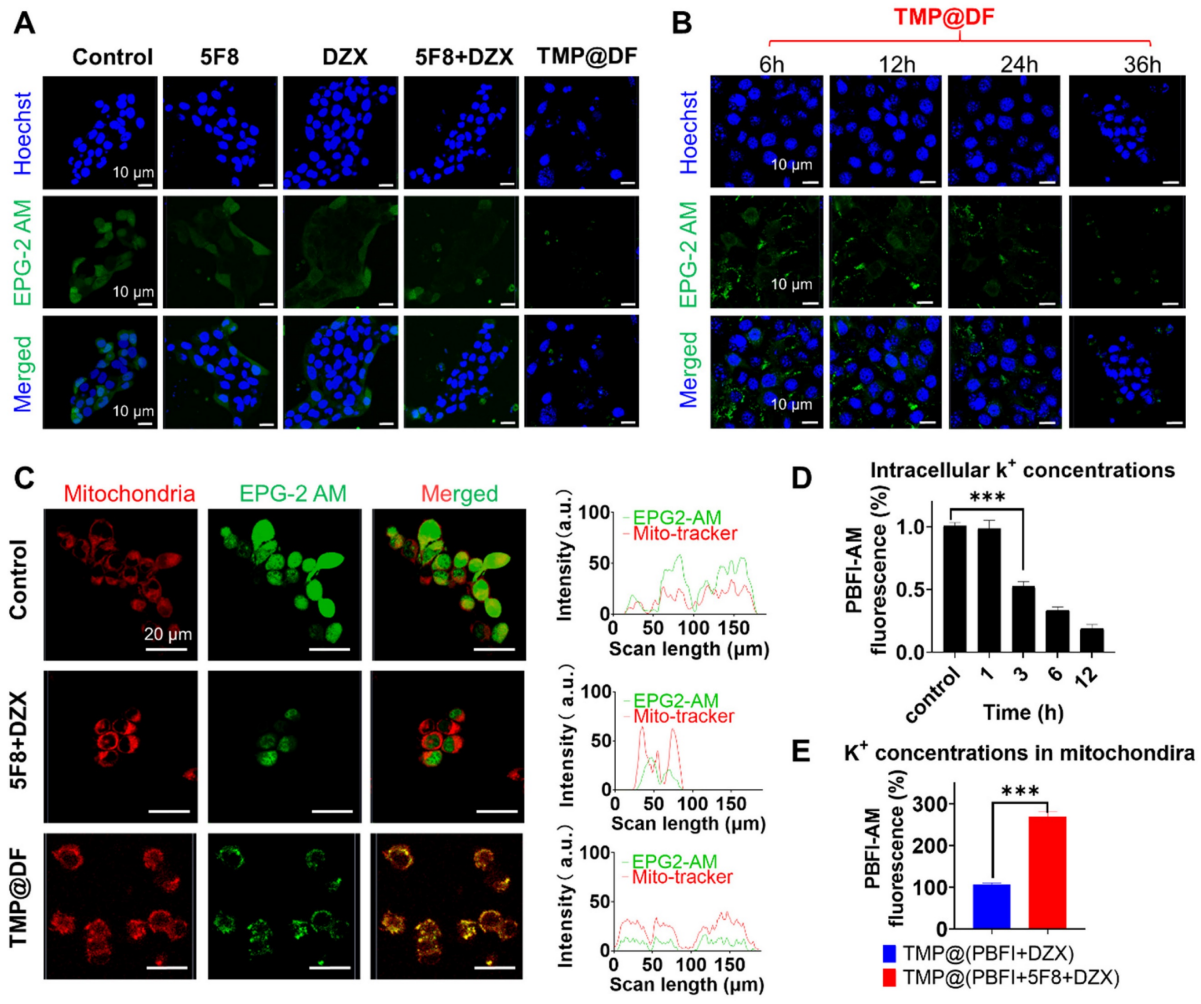
Changes of endogenous potassium ion level. (A) Fluorescence images of K^+^ changes in 4T1 cells treated with free 5F8, free DZX, 5F8+DZX and TMP@DF were detected with EPG-2 AM. (B) Confocal fluorescence images of K^+^ changes in 4T1 cells treated with TMP@DF NPs at different times measured by EPG-2 AM. (C) Fluorescence images of mitochondria co-localized with K^+^ after 5F8+DZX and TMP@DF treatment. (D) Fluorescence absorption of the PBFI-AM probe was used to detect K^+^ concentration in the cytoplasm of 4T1 cells. (E) Measurement of K^+^ concentration in the mitochondria of 4T1 cells using mitochondria-targeted co-delivery NPs of the PBFI-AM probe. Data are presented as means ± SD from three independent experiments. Statistical comparisons were analyzed by One-way ANOVA Tukey's multiple comparisons test. ^*^p < 0.05, ^**^p < 0.01, ^***^p < 0.001, n.s., not significant.

To further evaluate the effect of the construction of targeted artificial K^+^ selective ion channels on K^+^ homeostasis in the mitochondria of cancer cells, we investigated the changes in K^+^ concentration in the cytoplasm and mitochondria of cancer cells using green fluorescent K^+^ probes (EPG-2 AM) and PBFI-AM. As shown in **Figure 5A**, the green fluorescence in the TMP@DF group was significantly reduced compared with normal cells and the other groups, which indicated that the concentration of potassium ions in the cytoplasm was significantly reduced. Furthermore, we validated the effect of the intracellular potassium ion concentrations after treatment with TMP@DF NPs at different times (**Figure 5B**), including 6 h, 12 h, 24 h, and 36 h. As expected, with increasing duration of TMP@DF NPs action, intracellular potassium ions were significantly reduced until they were undetectable. This is consistent with the results of intracellular ROS generation at different times. It suggests that the intracellular ROS generation is due to the decrease of potassium ions leading to a change in potassium ion homeostasis, which results in oxidative stress. To more intuitively verify that the construction of mitochondria-targeted co-delivery artificial ion channels and activator molecule of mitoK_ATP_ caused the increase of K^+^ concentration in mitochondria, we further observed using laser confocal microscopy. As can be seen from **Figure 5C**, compared with other groups, more potassium ions could be obviously seen in the TMP@DF group entering the mitochondria, showing more green fluorescence (EPG-2 AM) and red fluorescence (mitochondrial localization dye) overlap. This finding also affirms that mitochondrial swelling is induced by an elevation in potassium ion concentration.

To better evaluate the change in intracellular potassium ion concentration, we further used potassium-binding benzofuran isophthalate-AM (PBFI-AM) as a probe to directly evaluate the change of intracellular potassium ion concentration with the increase in sample treatment time. It can be seen from **Figure 5D** that, the K^+^ in the cells of the TMP@DF group decreased significantly with the increase of treatment time. We hypothesized that this might be due to the establishment of mitochondrial potassium ion channels and the opening of the endogenous mitoK_ATP_ by DZX, which leads to the inflow of intracellular K^+^ into mitochondria according to the concentration gradient to break the ion homeostasis of mitochondria, thereby damaging the structure and function of mitochondria, and then causing cell apoptosis and resulting in a large loss of K^+^ from the cytoplasm 44, 45. To verify our guess, we further evaluated the changes of potassium ion concentration in mitochondria. As shown in** Figure 5E**, compared with the group with DZX alone (TMP@PBFI+DZX), the mitochondrial-targeted co-delivery 5F8 and DZX group (TMP@PBFI+5F8+DZX) showed higher potassium ion concentration. To be expected, by constructing artificial K^+^ channels in the mitochondrial membrane, TMP@DF activated endogenous potassium channels (mitoK_ATP_) and mediated a large K^+^ influx from the cytoplasm into the mitochondria, which severely interfered with the potassium ion homeostasis in the mitochondria and consequently triggered mitochondria-mediated apoptosis, which would provide a possibility to overcome the recurrence and metastasis of cancer cells.

### Synergy of 5F8 and DZX via mitochondria targeting on inhibiting postoperative tumor recurrence and metastasis *in vivo*

**Figure 6:**
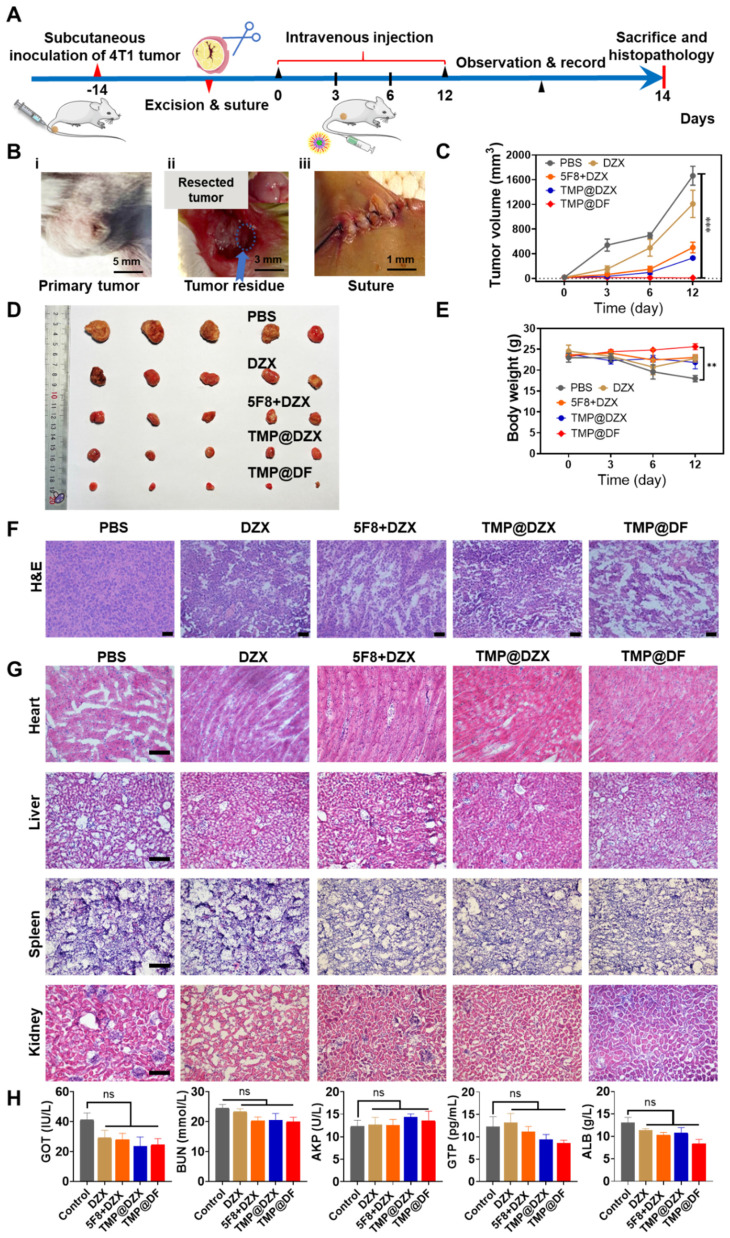
The synergy of mitochondria-targeted loaded 5F8 and DZX against postoperative 4T1 tumor recurrence. (A) Schematic diagram of experimental procedure involving establishing 4T1 tumor-bearing mouse model, excising tumors and harvesting samples. (B) The process of the surgery according to (A), including (i) tumor volume reached to more than 200 mm^3^, (ii) tumor resection, (iii) suture. (C) Average tumor growth curve of different groups (the statistical significance was acquired by one way ANOVA, ^***^p < 0.001). (D) Photograph of tumors removed from mice in different treatment groups. (E) Average body weight of mice in each group changing with time. (F) Microscopic images of H&E staining of the representative tumor sections. (G) H&E staining of main organs. (H) Serum biochemical analysis in different groups.

Tumor recurrence and metastasis are commonplace in postsurgical therapy, mainly attributed to incomplete tumor depletion, including the side effects of low therapeutic efficiency, inability to remove apoptotic-tolerant tumor cells and vast tumor necrosis. Fortunately, the intriguingly significant *in vitro* therapeutic effect of mitochondria-targeting loaded with 5F8 and DZX encouraged us to further evaluate their *in vivo* antitumor efficacy, which might hold promise in the postsurgical therapy and thereby achieve complete tumor cure. Thereby, a subcutaneous breast cancer recurrence model was established to verify the potential synergistic therapeutic effects of this formulation. As illustrated in **Figure 6A**, a postsurgical model of 4T1 tumor was established using female Balb/c mice and investigated the inhibition of tumor recurrence after surgery. In details, in order to mimic the situation of incomplete tumor removal in clinic, 4T1 breast tumor cells were inoculated into the flanks of mice, and about 90% of the tumor was surgically resected when the tumor volume exceed 200 mm^3^ (**Figure 6B**). The mice were randomly grouped, injected intravenously with different sample solutions at different times. During the observation, the tumor volume and body weight of each mouse were monitored. At the endpoint, all mice were sacrificed, and the samples were harvested for subsequent study. As shown in **Figure 6C-D**, the changes of tumor volumes for all groups are presented. It is shown that the TMP@DF NPs showed significant effect for the best suppression of tumor recurrence for postoperative tumor therapy. In accordance with the synergistic anti-cancer effects in vitro, the average tumor volume of mice in the TMP@DF group was lower than the average tumor volumes in other groups. This finding was more pronounced with increasing the treatment duration. The animals treated with TMP@DF NPs showed no significant body weight changes (**Figure 6E**), indicating that these mitochondria-targeted polymeric NPs are safe and reliable. This is probably attributed to the fact that TMP@DF NPs constructs a special ion channel in the mitochondrial inner membrane, meanwhile opening the endogenous mitoK_ATP_, which synergistically transports potassium ions. But in the free DZX group and 5F8 combine with DZX group, low cellular uptake resulted in a weak cell membrane permeability and a small role in causing apoptosis in tumor cells. In contrast, mitochondria-targeted NPs specifically delivered DZX to the mitochondrial membrane to turn on mitoK_ATP_. However, the limited mitoK_ATP_ in the mitochondrial inner membrane restrict potassium ion transport. Most interesting, due to the high electrochemical gradient of K^+^ inside and outside the mitochondrial membrane, K^+^ flows from the cytoplasm to the matrix. The K^+^ balance in the matrix is disturbed, which in turn generates ROS stress and causes mitochondrial damage, leading to enhanced antitumor effects and suppressed tumor recurrence. The suppression of postoperative tumor recurrence effect of mitochondria-targeted polymeric NPs was investigated by histological analysis of tumor tissues and major organs by H&E staining. In this regard, the purple color represents the nucleus and the red color represents the cytoplasm, as shown in **Figure 6F-G**. It was observed from **Figure 6F** that the nuclei of tumor tissues in the TMP@DF group were significantly less than those in other groups. The TMP@DF group showed more apoptotic regions in the tissues, indicating a better suppression of postsurgical tumor recurrence effect. In addition, there was no significant difference of morphology between major organs from different formulations, indicating that TMP@DF NPs did not cause significant damage to major organs, adequately demonstrating its excellent biocompatibility and biosafety.

To sufficiently evaluate the safety of mitochondria-targeted NPs, which is the top priority for treatment in the clinic, the biochemical analyses of urea nitrogen (BUN), alkaline phosphatase (AKP), albumin (ALB) and guanosine triphosphate (GTP) in the serum after intravenous injection were performed (**Figure 6H**). There was no significant difference among the index levels of all groups, which was consistent with the histological findings. Furthermore, we also measured the changes in blood potassium ion concentration within 15 days of administration (**Figure S8**), and there was no significant difference in the blood potassium ion concentration between the administration group and the control group. Therefore, the postoperative adjuvant therapy with mitochondria-targeted loaded 5F8 and DZX demonstrated decent biosecurity. These phenomena fully demonstrated that the synergy of mitochondria-targeted loaded 5F8 and DZX could effectively inhibit postoperative tumor recurrence *in vivo*.

**Figure 7:**
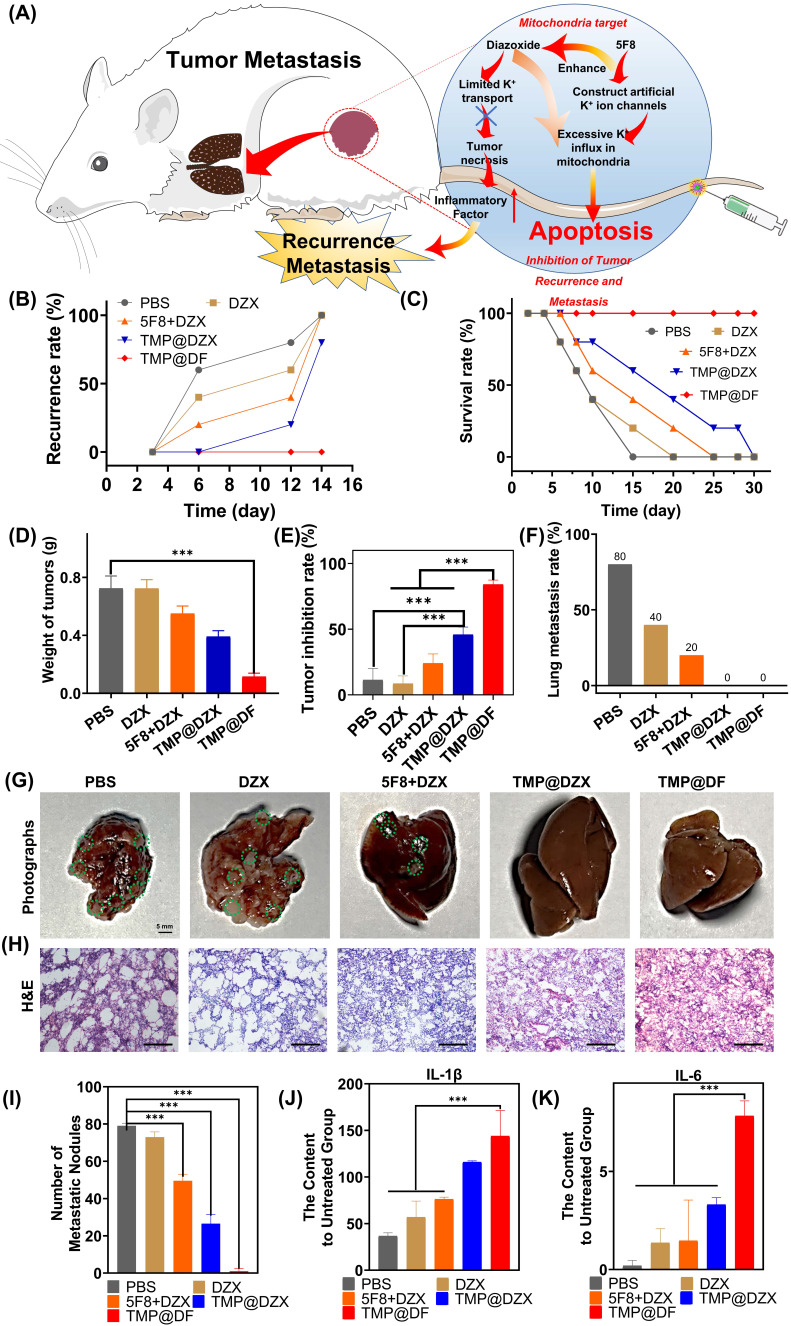
(A) Schematic illustration of TMP@DF NPs to inhibit tumor recurrence and metastasis *in vivo*. (B) Local recurrence rate, (C) survival rate and (D) weight of recurrent solid tumors from different treatment groups and tumor inhibition rate (E) on the 12th day. (F) The lung metastasis rate. (G) The photographs of lung and relevant images of lung section stained with H&E (H) in different groups, scale bar 100 μm. (I) The number of metastatic nodules in lung from different groups. (J-K) The content of IL-1β and IL-6 in lung tissue of mice after treatment. (The statistical significance was acquired by one way ANOVA, ^***^p < 0.001).

To further verify the effect of suppression postsurgical tumor recurrence and metastasis of TMP@DF NPs, in this study, the synergy of mitochondria-targeted loaded 5F8 and DZX could efficiently kill residual postsurgical tumor and apoptosis-resistant tumor cells to ensure less tumor metastasis (**Figure 7A**). As observed in **Figure 7B**, within 12 days, *in situ* recurrent tumors were found in all the mice in control group without any treatment. As expected, the *in situ* recurrence rate of mice in the TMP@DF NPs group was significantly decreased, and there was almost no recurrence compared to control and other groups. And the tumor weight of the TMP@DF group was much smaller than of other groups after 12 d (**Figure 7D**). Meanwhile, TMP@DF NPs group showed a higher survival rate compared with other groups (**Figure 7C**). A similar therapeutic synergy was also found in the inhibition rate of recurrence tumor weight obtained the treatment for 12 days (**Figure 7E**). It is exciting to find that TMP@DF NPs could efficiently disrupt potassium ion homeostasis of mitochondrial, further significantly decreasing the lung metastasis (**Figure 7F**). As observed in **Figure 7G-H**, 4T1 cells rapidly metastasized from the primary tumor to lung in control group, while the tumor metastasis was inhibited to varying degrees in other groups, and almost no metastatic nodule was observed in the mitochondria-targeted loaded with 5F8 and DZX group (**Figure 7I**). These sections of lung tissue were also observed in the H&E group. In addition, we found a large number of tumor cells were observed in the untreated group, suggesting a continual deterioration in the metastatic tumor. Tumor cells are aggressive following recurrence, and poorer IL-1β and IL-6 cytokine activity increases the likelihood of tumor lung tissue metastasis. Conversely, stronger IL-1β and IL-6 cytokine activity reduces the likelihood of lung tissue tumor metastasis. The content of IL-1β and IL-6 were tested in the tissue of lung of mice at the end of day 14 and were as shown in **Figure 7J-K**. The aforementioned results indicated that the synergy of mitochondria-targeted interfering potassium ion homeostasis strategy possessed a superior antitumor activity and could efficiently inhibit tumor recurrence and metastasis.

## Conclusions

In conclusion, on the basis of mitochondria-targeting of polymer (TMP) as a carrier, we designed and validated a synergy of mitochondria-targeted loaded 5F8 and DZX to suppress postoperative tumor recurrence and metastasis. The efficient anti-cancer therapy was achieved by the synergetic biochemical processes as follows. First and most importantly, 5F8 constructed artificial potassium ion channels in the inner mitochondrial membrane of cancer cells, while DZX opened endogenous mitoK_ATP_, which not only caused a large amount of cytoplasmic K^+^ to flood into mitochondria, but also disturbed the homeostasis of potassium ions in mitochondria. Secondly, oxidative stress was stimulated to generate ROS, which consumed the GSH and inhibited the expression of GPX4 proteins. Thirdly, the mitochondrial function and structure was damaged, including down-regulation ATP synthesis, decreased membrane potential of mitochondrial, mitochondrial swelling and membrane rupture, and then activation of the mitochondrial-associated apoptotic pathway. Besides the anti-cancer synergy, mitochondrial-targeted co-delivery 5F8 and DZX NPs interfered with the energy supply for tumor progression and tumor metastasis pathways. Based on the synergistic effect of natural and artificial ion channels, we developed a cancer strategy to inhibit tumor growth and metastasis by interfering with mitochondrial ion homeostasis, which will offer an applicable therapeutic approach to achieve the intended surgical effect of tumor resections and protect against tumor recurrence and metastasis.

## Materials and Methods

### Materials

According to earlier research, artificial K^+^-channel (5F8, *M_n_*=570.33) powder was created 31. It was purchased from Shanghai Aladdin Biochemical Technology Co., Ltd. Diazoxide (DZX, *M_n_*=230.67) powder. From Sigma-Aldrich, we received branching polyethyleneimine (PEI, *M_n_*=25 kDa), methoxy polyethylene glycol (MPEG, molecular weight 5 kDa), 1,1'-carboxyimidazole (CDI, >97%), toluene (99.8%), N,N-dimethylformamide (99.8%), and dimethyl sulfoxide (99.9%). Energy Chemical provided the 5-carboxy pentyl triphenyl phosphonium bromide (TPP-C_5_-COOH, 98%). Genbio provided N-Hydroxysuccinimide (NHS, 99%). Mucklin supplied the 1-ethyl-3-dimethylaminopropyl carbodiimide hydrochloride (EDC, 98.5%). Thermo Fisher Scientific provided the phosphate buffered saline (PBS) and Duchenne modified Eagle medium (DMEM). Dibutyltin dilaurate and 1,6-Hexamethylene diisocyanate (HMDI) were purchased from Sigma-Aldrich. Shanghai Aladdin Biochemical Technology Co., Ltd. supplied the thiazolyl blue tetrazolium bromide (MTT). Reduced L-glutathione (GSH) was purchased from Macklin (Shanghai, China). A cell apoptosis kit containing FITC and Annexin-PI was provided by Yeasen (Shanghai, China). Chloral hydrate and dimethyl sulfoxide (DMSO) were supplied by Sinopharm Chemical Reagent Co., Ltd. Eisen Biotech (Shanghai) Co., Ltd. provided the antibiotics and the trypsin. Solarbio provided the H&E staining kit and the Masson trichrome staining kit. We purchased from Biotec Biotech optimal cutting temperature compound (OCT), encapsulant, and sealer (containing DAPI). Balb/c nude mice were provided by the Shanghai-based Slac Laboratory Animal Company.

### Synthesis of MPEG-PCL-PEI-TPP (TMP) copolymer

An earlier report on the MPEG-PCL-PEI (MPP) copolymer's synthesis can be found here 42, 43. Amidation reaction between MPP and TPP produced TMP 46. More specifically, the MPP was dissolved in DMSO to prepare a solution with a concentration of 1 mmol/L as solution (1); dissolved 6 mmol/L of TPP-C_5_-COOH in DMSO, and then sequentially activated by adding 6 mmol/L of EDC and 6 mmol/L of NHS with a reaction time of 30 min as solution (2); the solution (1) was added slowly to (2) and reaction was carried out for 12 h at room temperature, to be purified by dialysis, in which the molecular weight of the dialysis bag was 3400 D and the dialysis time was 2 days; the final product was acquired by lyophilization.

### Characterization of TMP copolymer

The structural characteristics of the molecules in the copolymer at room temperature were determined by ^31^P NMR spectroscopy (400 MHz, Bruker, Switzerland) with CDCl_3_ as the solvent.

### Cell culture

The 4T1 cells were cultured in DMEM with 10% FBS and 1% penicillin and streptomycin in a humidified incubator containing 5% CO_2_ and maintain at 37 °C.

### Cytotoxicity evaluation

To examine how various formulation samples affected tumor cell proliferation, the MTT method was employed. The Chinese Academy of Sciences Cell Bank provided 293T (human embryonic kidney cells) and 4T1 (mouse breast cancer cells). A humidified environment with 5% CO_2_ at 37 °C was used to cultivate the cells in Dulbecco's modified Eagle's medium (DMEM), and all of them received an addition of 10% fetal bovine serum (FBS) and 100 U/mL penicillin. A density of 5 × 10^3^ cells per well was used to seed the specified cells in 96-well plates. The medium was discarded after 24 hours of incubation at 37 °C, and 100 μL of solutions containing various concentrations of the tested samples' serum-free media were then added. Incubation at 37 °C was then continued. Each well received 10 μL of 3-(4,5-dimethyl-2-thiazolyl)-2,5-diphenyl-2-H-tetrazole (MTT) after 24 hours, which was added and incubated for an additional 4 hours at 37 °C. In order to dissolve the crystals, 150 μL of DMSO were added to each well in place of the supernatant. Finally, the absorbance at 492 nm was used to determine the cell viability.

### Cellular uptake of copolymers

At a density of 5 × 10^4^ cells per well, the cells were planted in 12-well plates and cultivated for 24 hours. Freshly made multimeric non-serum medium solutions containing TMP were applied to the specified cells for 24 hours. Phosphate-buffered saline (PBS, 0.1 M, pH = 7.4) solution was used to wash and resuscitate the cells after they had been collected at 0 h, 1 h, 4 h, 12 h, and 24 h. Flow cytometry measurements were used to evaluate the cells' fluorescence.

### Lysosomal escape ability of copolymer

The drug vectors undergoing post-cellularization must allow lysosomal escape in order to properly transport medicines to mitochondrial targets. With the help of FITC (fluorescein isothiocyanate), MPP and TMP were labelled.

The cells were planted in a 24-well plate at a density of 2 × 10^4^ cells per well until they adhered to the wall. The solution containing TMP-FITC was then added, and the cells were incubated at 37 °C in an incubator with 5% CO_2_ for 1 h, 3 h, 6 h, and 12 h. To get rid of the copolymer solution and other contaminants that had been adsorbed on the surface of the cells, PBS was applied three times to the cells. The slides were sealed after being treated in a 4% formaldehyde solution for 15 minutes. Confocal laser scanning microscopy (CLSM) was used for observing and taking images of the fluorescent lysosomal escape of the fluorescently labelled copolymer in the cells.

### Mitochondrial targeting ability of copolymer

The appropriate cells were infused into 24-well plates at a density of 20,000 cells per well until the cells had adhered to the wall. MPP-FITC and TMP-FITC solutions were then added, and the incubator was preheated for 24 hours. The culture media was taken out, and Mitotracker Red (100 nM) was added. The medium was to be stained for 30 minutes. After three rounds of washing with PBS, the cells were fixed in 4% formaldehyde solution for 15 minutes before being sealed in glycerol. By using CLSM, it was possible to see how the fluorescently labelled copolymer was co-localized within the mitochondria of the cells, and fluorescent images were taken. Semi-quantitative examination of the co-localization coefficients of the vector and mitochondria was performed using ImageJ software.

### Fabrication and characterization of drug loaded TMP@DF complex

To begin with, 5F8, DZX and TMP were dissolved in anhydrous ethanol; followed by mixing 5F8 with DZX ethanol solution (1:1 m/m) as solution ①, then solution ① was mixed with TMP ethanol solution (1:5 m/m) and added dropwise to deionized H_2_O (1 mL) under ultrasonic stirring, the concentrations of 5F8, DZX and polymer in the prepared solutions were 1.75 μg/mL, 1.75 μg/mL and 17.5 μg/mL, respectively, and the sonication was continued for 30 min; eventually, the solution was stirred overnight and the organic solvent was evaporated. The obtained complex (TMP@DF) was vacuum dried and storage at -20 °C before use.

### Characterization of polymeric nanoparticles

Double-distilled water was used to dissolve TMP and TMP@DF at a concentration of 0.5 mg/mL. Dynamic light scattering (DLS) using a Zetasizer (Malvern, Nano-ZS90) was used to characterize the hydration size, and stability of the produced nanoparticles. The morphology and particle size of the nanoparticles were examined using a transmission electron microscope (TEM) (Zeiss 500).

### Measurement of drug release *in vitro*

The solubility of free 5F8 and DZX in pH 7.4 phosphate buffered saline was evaluated independently at 37 °C to examine the drug release from 5F8 and DZX loaded micelles. In pH 7.4 PBS, extra amounts of 5F8 and DZX were added while shaking horizontally at 60 rpm and 37 °C. Supernatants were collected after 72 hours, high performance liquid chromatography (HPLC) was used to quantify the amounts of 5F8 and DZX individually.

By dialysis of 5F8 and DZX loaded micellar solutions in pH 7.4 phosphate buffered saline using a shaker at 37 °C with horizontal shaking (60 rpm), *in vitro* drug release characteristics were investigated. Dialyzing the 1.0 mL of TMP@DF micelle solution against 20.0 mL of buffer (MWCO: 3.5 kDa). At specified intervals, new buffer was added to replace any remaining media outside of the dialysis tube. HPLC was used to find the 5F8 and DZX levels in all samples.

### Evaluation of antitumor activity in cells

To investigate the susceptibility of tumor cells to polymeric nanoparticles with various mass ratios, 4T1 cells were screened. In 96-well plates, a certain number of cells were evenly placed before the plates were incubated for 24 hours in the incubator. Following incubation, the media was changed, and the cells were exposed to successively higher concentrations of the tested substances for subsequent culture. Cell counting kit (CCK-8) reagent was added after 48 hours of treatment for a further 4 hours of culture at 37 °C. The enzyme marker was used to detect the absorbance at 450 nm in each well, and the survival rate CV% of the cells in each experimental group was determined using the formula below:

CV%=(A_t_/A_c_) ×100%

where CV% stands for cell survival rate; A_t_ represents the absorbance value of the experimental group's cells; and A_c_ displays the value of the control group's inert cell population.

### The level of ROS in cancer cells treated with NPs

2,7-Dichlorodihydrofluorescein diacetate (DCFH-DA) probe was used to measure the levels of intracellular reactive oxygen species (ROS). Using 2'-[4-ethoxyphenyl]-5-[4-methyl-1-piperazinyl]-2,5'-bi-1H-benzimidazole trihydrate (Hoechst 33342) allowed researchers to visualise the nucleus. The 12 well plates with the well-grown 4T1 cells were incubated for 24 hours. After incubation, they were switched to serum-free media that had been diluted with nanoparticles of TMP@DZX (containing DZX at a concentration of 3.5 μg/mL) and TMP@DF (containing 5F8 and DZX at a concentration of 1.75 μg/mL, respectively) and continue incubating for 3, 6 and 12 hours, respectively. The intracellular levels of ROS were then collected and examined qualitatively and quantitatively by CLSM, after the cells had been treated with DCFH-DA probe at a concentration of 10 μM and incubated for 30 min at 37 °C, away from light.

### Detection of apoptosis and western blotting

The cells were inoculated in 12-well plates with 100,000 cells per well. After 24 hours of incubation, the cells were treated with TMP, free DZX (3.5 μg/mL), free 5F8+DZX (1.75 μg/mL, respectively), TMP@DZX (containing DZX 3.5 μg/mL) and TMP@DF (containing 5F8 and DZX, 1.75 μg/mL, respectively) and incubated for 12 hours. Following the manufacturer's instructions, flow cytometry was used to quantify apoptosis using the annexin V-Fluorescein Isothiocyanate (FITC)/Propidium Iodide (PI) Apoptosis Detection Kit. In a nutshell, trypsinized cells were centrifuged at 1000 g for 10 min, after being trypsinized with 0.30% trypsin. PBS was used to wash the cell pellet. They were reconstituted in 200 L of annexin V-FITC containing binding buffer. In order to analyse apoptotic cells, flow cytometry was used to analyse 5 L of each V-FITC (10 μg/mL) and PI (5 μg/mL) for 15 min at room temperature in the dark. This was followed by FlowJo VX software. The following tests were done on the protein levels of 4T1 cells. The cell lysates were electrophoresed on 8% SDS-PAGE and then transferred to PVDF membranes after the cells had been collected and lysed in RIPA lysis buffer. Membranes were pre-incubated with the primary antibody in 5% BSA in TBST overnight at 4 °C after being blocked with 5% milk in TBST (10 mM Tris-HCl, pH 8.0, 150 mM NaCl, 0.1% Tween 20) for 1 hour. Membranes were rinsed three times with TBST before being incubated for 1 hour at room temperature with TBST that had anti-mouse or anti-rabbit IgG that had been HRP-conjugated. The ECL system detected the immuno-reactive bands following three TBST washes.

### Intracellular mitochondrial membrane potential (Δφ m) assay (JC-1)

The cationic fluorescent probe JC-1 was used to evaluate the mitochondrial membrane potential (Δφ m). The strength of the transition from red to green fluorescence was used to gauge changes in mitochondrial depolarization. 50,000 cells/well of 4T1 cells were cultured on 12-well plates. 24 h later, TMP, free DZX (3.5 μg/mL), free 5F8+DZX (1.75 μg/mL, respectively), TMP@DZX (containing DZX 3.5 μg/mL) and copolymer nanoparticles TMP@DF (containing 5F8 and DZX, 1.75 μg/mL, respectively) were added and incubated for 2 h, 6 h and 12 h. The cells were incubated with 5 μg/mL of JC-1 staining solution at 37 °C for 30 min protected from light. Fluorescence photographs were taken with CLSM and recorded.

### Mitochondrial ultrastructure

Cells were exposed to TMP@DF polymer nanoparticles that contained 1.75 μg/mL of 5F8 and 1.75 μg/mL of DZX. Blank media was applied as the control treatment. Following a 12-hour incubation period, the cells were washed three times with PBS, fixed for two to three hours with a working solution of 2.5% glutaraldehyde, diluted twice as much with phosphate buffer, and then refrigerated at 4 °C. The apposed fixed cells were then scraped off with a cell scraper, the phosphate buffer was removed, a phosphate buffer containing 5% bovine serum albumin (BSA) was added, the cells were collected, and the mitochondrial ultrastructure in the cells was examined under a transmission electron microscope.

### Experiment for cellular GSH

4T1 tumor cells were seeded in 6-well plates and cultured at 37 °C for 24 hours before being exposed to various substances. After that, cells were removed and repeatedly cleaned with PBS. Following cell lysis with Triton-X-100 lysis solution, the quantity of GSH was measured using a GSH assay kit. Based on a comparison to the GSH content of untreated cells, the percentage of GSH in cells was estimated.

### Caspase activity

Peptide substrates that fluoresce when cleaved by particular caspases can be used to evaluate the activity of Caspase 3 and Caspase 9 in cells. To summarize, tumor cells were incubated with TMP, free DZX (3.5 μg/mL), free 5F8+DZX (1.75 μg/mL, respectively), TMP@DZX (containing DZX 3.5 μg/mL) and TMP@DF (containing 5F8 and DZX, 1.75 μg/mL, respectively), using blank medium as a control. After 24 hours, cells were collected and lysed. Centrifugation of cell lysates at 12,000 rpm for 10 minutes at 4 °C took place. Substrates for Caspase 3 and Caspase 9 were applied to the supernatant after storage. An enzyme marker was used to quantify Caspase 3 and Caspase 9 activities at 405 nm, and activity ratios were determined in accordance with the guidelines.

### Synergetic tumor cytotoxicity of 5F8 and DZX in mitochondrial of cancer cells and synergism calculation

The network application Synergy Finder https://synergyfinder.fimm.fi) (accessed on 20 February 2023), which can be used for interactive analysis and visualization of synergistic medication combinations in preclinical model systems, was used to determine the synergy score of the synthetic potassium channel (5F8) and DZX with mitochondrial targeting in cancer cells. In mitochondria-targeted copolymer nanoparticles (TMP@DF), the mass concentration ratios of loaded 5F8 to DZX were 0:1, 0.3:1, 0.5:1, 1:1, and 2:1, respectively. The survival of cells screening statistics were used to analyse the combinatorial effects of the aforementioned TMP@DF, and the results were subsequently submitted to Synergy Finder to accurately determine the synergistic or inhibitory effects. The synergy score was computed using the zero interaction (ZIP) potency model. When the synergy score was less than -10, it was presumed that the pharmacological interaction was hostile. The likelihood of the medications being additive increases when the value is between -10 and 10. The interaction is anticipated to be synergistic when the synergistic fraction is greater than 10 (https://synergyfinder.fimm.fi).

### Intracellular ATP level measurement

We utilized an ATP luminescence test kit (S0026) to investigate the change in intracellular ATP. 4T1 cells were planted into a 48-well plate at a density of 2 × 10^5^ cells/well until the cells had adhered to the wall. TMP, free DZX (3.5 μg/mL), free 5F8+DZX (1.75 μg/mL, respectively), TMP@DZX (containing DZX 3.5 μg/mL) and TMP@DF (containing 5F8 and DZX, 1.75 μg/mL, respectively) solutions were then added, respectively, and the cells were incubated at 37 °C in an incubator with 5% CO_2_. Cells were retrieved 12 hours later, and the suggested method was used to measure the intracellular ATP level. A Promega GloMax luminometer (GloMax Discover, The USA) was used to measure the ATP luminescent signals, and calibration curves were used to determine the ATP level.

### *In vitro* potassium concentration evaluation

After being cultured for 24 hours in a 24-well plate, 4T1 cells were treated with free 5F8 (3.5 μg/mL), DZX (3.5 μg/mL), free 5F8+DZX (1.75 μg/mL, respectively), and TMP@DF (containing 5F8 and DZX, 1.75 μg/mL, respectively). All groups were given the Enhanced Potassium Green-2 AM (EPG-2 AM) probe and 2'-[4-ethoxyphenyl]-5-[4-methyl-1-piperazinyl]-2,5'-bi-1H-benzimidazole trihydrate (Hoechst 33342) for 30 min after 24 hours of incubation. Slices were sealed by fixation with 4% paraformaldehyde for 15 minutes. CLSM was used to capture fluorescent images. Additionally, utilizing a fluorescent probe that binds to potassium utilizing benzofuran isophthalate-AM (PBFI-AM), the amounts of potassium ions inside of cells and mitochondria were also assessed.

### *In vivo* therapeutic efficacy for antitumor postsurgical recurrence and metastasis

Balb/c female mice weighing 20-30 g were utilized in the investigation to properly comprehend the relevance of TMP@DF NPs (containing 5F8 and DZX, 0.15 mg/30 g, respectively) for the treatment of cancer postoperative recurrence and metastasis. Prior to the experiment, all mice were kept in typical cages for a week to get acquainted to the lab setting. They were given free access to food and water during the trial. Each Balb/c mouse was subcutaneously injected with 4T1 cells suspended in PBS to create the tumor model. On day 14 following tumor injection, the tumor volume was approximately 200 mm^3^. Following that, each tumor had at least 90% of its volume removed, mimicking the positive margins in clinical surgery. After suturing the site where the tumor was excised, the mice were administered the drug via intravenous injection. The following equation was used to determine the tumor volume. V_tumor_ (mm^3^) = Length_tumor_ (mm) × width_tumor_^2^ (mm^2^)/2. Throughout the course of treatment, each mouse's tumor volume and body weight were monitored. Calculation of the tumor inhibition rate of each group was done using the following formula: Tumor inhibition rate = (W_tumor, control_-W_tumor, x_)/W_tumor, control_ ×100%, where W_tumor, control_ signifies the average W_tumor_ of mice in the control group, and W_tumor, x_ indicates the W_tumor_ of each mouse in x group. The Xiamen University Animal Care and Use Committee (XMULAC20210092) approved the animal experiments, and the National Institutes of Health's standards were followed in the evaluation of the animal research. Certain humane outcomes, such as tumor volumes more than 1000 mm^3^, ulcerations, necrosis, or infections, were examined for viability while taking animal welfare and ethical concerns into account. Tumors were taken out at the conclusion of the *in vivo* tests, and pictures were taken.

For histological analysis, tissues from heart, liver, spleen, lung, and kidney were harvested and soaked in 15% and 30% sucrose solution for 24 h and 12 h, respectively. Thereafter, tissue sections were embedded in an optimal cutting temperature (OCT) compound and sliced into tissue sections with a thickness of 6 μm by using a freeze slicer (CM1900, Leica, Germany). Then, they were stained with hematoxylin & eosin (H&E) for observation and analysis under a microscope.

### Evaluation of *in vivo* biosafety

Throughout the *in vivo* experiments, the mice's weight is tracked, and biochemical markers such kidney (BUN and CRE) and liver (ALP, ALT, AST) function are examined. At the end of the *in vivo* experiment, mouse serum was collected and then combined in a heparin sodium anticoagulant vessel tube for testing. The following were utilized to determine liver function: alanine aminotransferase (ALT), aspartate aminotransferase (AST), total bilirubin level (TBIL), and total protein (TP). To test kidney function, the levels of blood urea nitrogen (BUN) and creatinine (CRE) were measured. The amounts of the suitable biomarkers were determined after all tests were assessed using biochemical factor kits in line with pre-established sample curves.

### Research into tumor metastasis and recurrence

The tumor recurrence was examined over a 12-day treatment therapy. After 14 days, mice with cervical dislocation and the matching lung were harvested. The internal status was evaluated by H&E staining, and the number of surface metastatic nodules was counted.

### Enzyme-Linked Immunosorbent Assay (ELISA)

Using murine-specific IL-6 and IL-1β Quantikine ELISAs (both R&D Systems, Minneapolis, MN, USA), interleukin-6 (IL-6) and interleukin-1β (IL-1β), concentrations in mouse lung tissue were assessed in accordance with the manufacturer's recommendations.

### Statistical analysis

Values are shown as mean ± standard deviation, a GraphPad Prism 5.0 software and ImageJ were applied for statistical analysis. Multiple t tests were used for statistical analysis to compare statistical significance. ^*^p < 0.05, ^**^p < 0.01 and ^***^p < 0.001 were respectively recognized as statistically significant, highly significant, and very significant.

## Supplementary Material

Supplementary methods and figures.

## References

[1] Feske S, Skolnik EY, Prakriya M (2012). Ion channels and transporters in lymphocyte function and immunity. Nat Rev Immunol.

[2] Prevarskaya N, Skryma R, Shuba Y (2010). Ion channels and the hallmarks of cancer. Trends Mol Med.

[3] Grissmer S (1997). Potassium channels still hot. Trends Pharmacol Sci.

[4] Breuer E-K, Fukushiro-Lopes D, Dalheim A, Burnette M, Zartman J, Kaja S (2019). Potassium channel activity controls breast cancer metastasis by affecting β-catenin signaling. Cell Death Discov.

[5] Wible BA, Wang L, Kuryshev YA, Basu A, Haldar S, Brown AM (2002). Increased K^+^ efflux and apoptosis induced by the potassium channel modulatory protein KChAP/PIAS3 in prostate cancer cells. J Biol Chem.

[6] Prevarskaya N, Skryma R, Bidaux G, Flourakis M, Shuba Y (2007). Ion channels in death and differentiation of prostate cancer cells. Cell Death Differ.

[7] Pardo LA, Stühmer W (2014). The roles of K^+^ channels in cancer. Nat Rev Cancer.

[8] Szabo I, Zoratti M, Biasutto L (2021). Targeting mitochondrial ion channels for cancer therapy. Redox Biol.

[9] Huber SM (2013). Oncochannels. Cell Calcium.

[10] Leanza L, Zoratti M, Gulbins E, Szabo I (2014). Mitochondrial ion channels as oncological targets. Oncogene.

[11] Urbani A, Prosdocimi E, Carrer A, Checchetto V, Szabò I (2021). Mitochondrial ion channels of the inner membrane and their regulation in cell death signaling. Front Cell Dev Biol.

[12] Wrzosek A, Augustynek B, Żochowska M, Szewczyk A (2020). Mitochondrial potassium channels as druggable targets. Biomolecules.

[13] Bonnet S, Archer SL, Allalunis-Turner J, Haromy A, Beaulieu C, Thompson R (2007). A mitochondria-K^+^ channel axis is suppressed in cancer and its normalization promotes apoptosis and inhibits cancer growth. Cancer Cell.

[14] Coetzee WA (2013). Multiplicity of effectors of the cardioprotective agent, diazoxide. Pharmacol Ther.

[15] D'hahan N, Moreau C, Prost A-L, Jacquet H, Alekseev AE, Terzic A (1999). Pharmacological plasticity of cardiac ATP-sensitive potassium channels toward diazoxide revealed by ADP. Proc Natl Acad Sci USA.

[16] Lee YS (2004). Mechanism of apoptosis induced by diazoxide, a K^+^ channel opener, in HepG2 Human hepatoma cells. Arch Pharm Res.

[17] Klenner T, Berger MR, Zelezny O, Fink M, Schmähl D (1990). Antineoplastic efficacy of melphalan and N-(2-chloroethyl)-N-nitrosocarbamoyl-ω-lysine, in combination with diazoxide or insulin in autochthonous mammary carcinoma of the Sprague-Dawley rat. J Cancer Res Clin.

[18] Ding J, Ge D, Guo W, Lu C (2009). Diazoxide-mediated growth inhibition in human lung cancer cells via downregulation of β-catenin-mediated cyclin D1 transcription. Lung.

[19] Núñez L, Valero RA, Senovilla L, Sanz-Blasco S, García-Sancho J, Villalobos C (2006). Cell proliferation depends on mitochondrial Ca^2+^ uptake: inhibition by salicylate. J Physiol.

[20] Dȩbska G, May R, Kicińska A, Szewczyk A, Elger CE, Kunz WS (2001). Potassium channel openers depolarize hippocampal mitochondria. Brain Res.

[21] Katoh H, Nishigaki N, Hayashi H (2002). Diazoxide opens the mitochondrial permeability transition pore and alters Ca^2+^ transients in rat ventricular myocytes. Circulation.

[22] Kowaltowski AJ, Seetharaman S, Paucek P, Garlid KD (2001). Bioenergetic consequences of opening the ATP-sensitive K^+^ channel of heart mitochondria. AmJ Physiol-Heart C.

[23] Grimmsmann T, Rustenbeck I (1998). Direct effects of diazoxide on mitochondria in pancreatic B-cells and on isolated liver mitochondria. Br J Pharmacol.

[24] Kim HK, Noh YH, Nilius B, Ko KS, Rhee BD, Kim N (2017). Current and upcoming mitochondrial targets for cancer therapy. Semin Cancer Biol.

[25] Remillard CV, Yuan JXJ (2004). Activation of K^+^ channels: an essential pathway in programmed cell death. Am J Physiol Lung Cell Mol Physiol.

[26] Zheng S-P, Huang L-B, Sun Z, Barboiu M (2021). Self-assembled artificial ion-channels toward natural selection of functions. Angew Chem Int Ed.

[27] Mayer M, Yang J (2013). Engineered ion channels as emerging tools for chemical biology. Acc Chem Res.

[28] Chen S, Wang Y, Nie T, Bao C, Wang C, Xu T (2018). An artificial molecular shuttle operates in lipid bilayers for ion transport. J Am Chem Soc.

[29] Saha T, Gautam A, Mukherjee A, Lahiri M, Talukdar P (2016). Chloride transport through supramolecular barrel-rosette ion channels: lipophilic control and apoptosis-inducing activity. J Am Chem Soc.

[30] Zhang H, Ye R, Mu Y, Li T, Zeng H (2021). Small molecule-based highly active and selective K^+^ transporters with potent anticancer activities. Nano Letters.

[31] Ren C, Shen J, Zeng H (2017). Combinatorial evolution of fast-conducting highly selective K^+^-channels via modularly tunable girectional assembly of crown ethers. J Am Chem Soc.

[32] Shen F-F, Dai S-Y, Wong N-K, Deng S, Wong AS-T, Yang D (2020). Mediating K^+^/H^+^ transport on organelle membranes to selectively eradicate cancer stem cells with a small molecule. J Am Chem Soc.

[33] Shen J, Gu Y, Ke L, Zhang Q, Cao Y, Lin Y (2022). Cholesterol-stabilized membrane-active nanopores with anticancer activities. Nat Commun.

[34] Ma P, Luo Z, Li Z, Lin Y, Li Z, Wu Z (2024). Mitochondrial artificial K^+^ channel construction using MPTPP@5F8 nanoparticles for overcoming cancer drug resistance via disrupting cellular ion homeostasis. Adv Healthc Mater.

[35] Dong S, Dong Y, Zhao Z, Liu J, Liu S, Feng L (2023). “Electron transport chain interference” strategy of amplified mild-photothermal therapy and defect-engineered multi-enzymatic activities for synergistic tumor-personalized suppression. J Am Chem Soc.

[36] Du Y, Yang J, He F, Zhao X, Zhou J, Zang P (2024). Revealing the mutually enhanced mechanism of necroptosis and immunotherapy induced by defect engineering and piezoelectric effect. Adv Mater.

[37] Yang L, Tian B, Xie Y, Dong S, Yang M, Gai S (2023). Oxygen-vacancy-rich piezoelectric BiO_2_-x nanosheets for augmented piezocatalytic, sonothermal, and enzymatic therapies. Adv Mater.

[38] Dong Y, Dong S, Yu C, Liu J, Gai S, Xie Y (2023). Mitochondria-targeting Cu_3_VS_4_ nanostructure with high copper ionic mobility for photothermoelectric therapy. Sci Adv.

[39] Dong Y, Dong S, Liu B, Yu C, Liu J, Yang D (2021). 2D piezoelectric Bi_2_MoO_6_ nanoribbons for GSH-enhanced sonodynamic therapy. Adv Mater.

[40] Zhao R, Zhu Y, Feng L, Liu B, Hu Y, Zhu H (2024). Architecture of vanadium-based MXene dysregulating tumor redox homeostasis for amplified nanozyme catalytic/photothermal therapy. Adv Mater.

[41] Zhu C, Ke L, Ao X, Chen Y, Cheng H, Xin H (2024). Injectable supramolecular hydrogels for in situ programming of Car-T cells toward solid tumor immunotherapy. Adv Mater.

[42] Li ZG, Fan XT, Luo Z, Loh XJ, Ma YD, Ye EY (2022). Nanoenzyme-chitosan hydrogel complex with cascade catalytic and self-reinforced antibacterial performance for accelerated healing of diabetic wounds. Nanoscale.

[43] Liu MT, Luo Z, Li ZG, Lai XY, Loh XJ, Wu CS (2023). Engineered celastrol and plasmid co-delivery for in situ expression and targeted mitochondrial relocation of Nur77 protein towards effective drug resistance reversion. Chem Eng J.

[44] Lee D, Lee SH, Noh I, Oh E, Ryu H, Ha J (2019). A helical polypeptide-based potassium ionophore induces endoplasmic reticulum stress-mediated apoptosis by perturbing ion homeostasis. Adv Sci.

[45] Checchetto V, Azzolini M, Peruzzo R, Capitanio P, Leanza L (2018). Mitochondrial potassium channels in cell death. Biochem Bioph Res Co.

[46] Zhang H, Pan J, Wang T, Lai Y, Liu X, Chen F (2022). Sequentially activatable polypeptide nanoparticles for combinatory photodynamic chemotherapy of breast Cancer. ACS Appl Mater Interfaces.

