# Supplementary material for: Combined activation of artificial and natural ion channels for disrupting mitochondrial ion homeostasis towards effective postoperative tumor recurrence and metastasis suppression
Source: Theranostics. 2024 May 27;14(8):3282–99. doi: 10.7150/thno.94855 (PMC11155400; doi:10.7150/thno.94855)
Supplement: Supplementary file 1 — Supplementary methods and figures. [file thnov14p3282s1.pdf]

Supporting Information

Combined activation of artificial and natural ion channels for disrupting mitochondrial ion homeostasis towards effective postoperative tumor recurrence and metastasis suppression

Panqin Ma[a,1], *Zheng Luo*[a,b,1], *Qi Wang*[a], *Ying Chen*[a], *Feng Liu*[b], *Changliang Ren*[a,d], *Caisheng Wu*[a,*], *Zibiao Li*[b,c,*], *Yun-Long Wu*[a,*]

[a] Fujian Provincial Key Laboratory of Innovative Drug Target Research and State Key Laboratory of Cellular Stress Biology, School of Pharmaceutical Sciences, Xiamen University, Xiamen 361102, China

[b] Institute of Materials Research and Engineering, A*STAR (Agency for Science, Technology and Research), 2 Fusionopolis Way, Innovis, #08-03, Singapore 138634, Singapore

[c] Institute of Sustainability for Chemicals, Energy and Environment (ISCE2), A*STAR (Agency for Science, Technology and Research), 1 Pesek Road, Jurong Island, Singapore, 627833, Singapore

[d] Shenzhen Research Institute of Xiamen University, Shenzhen, Guangdong 518057, China.

* Corresponding authors.

E-mail address: wuyl@xmu.edu.cn (Y.-L. Wu), lizb@imre.a-star.edu.sg (Z. Li), wucsh@xmu.edu.cn (C. Wu).

[1]The authors contributed equally to this work.

This PDF file includes:

Supplementary Experimental section

Figures. S1 to S8

Experimental section

Fabrication and characterization of drug loaded TMP@DF complex

To begin with, 5F8, DZX and TMP were dissolved in anhydrous ethanol; followed by mixing 5F8 with DZX ethanol solution (1:1 m/m) as solution ①, then solution ① was mixed with TMP ethanol solution (1:5 m/m) and added dropwise to deionized H_2O (1 mL) under ultrasonic stirring, the concentrations of 5F8, DZX and polymer in the prepared solutions were 1.75 μg/mL, 1.75 μg/mL and 17.5 μg/mL, respectively, and the sonication was continued for 30 min; eventually, the solution was stirred overnight and the organic solvent was evaporated. The obtained complex (TMP@DF) was vacuum dried and storage at -20 °C before use.

Evaluation of antitumor activity in cells

To investigate the susceptibility of tumor cells to polymeric nanoparticles with various mass ratios, 4T1 cells were screened. In 96-well plates, a certain number of cells were evenly placed before the plates were incubated for 24 hours in the incubator. Following incubation, the media was changed, and the cells were exposed to successively higher concentrations of the tested substances for subsequent culture. Cell counting kit (CCK-8) reagent was added after 48 hours of treatment for a further 4 hours of culture at 37 °C. The enzyme marker was used to detect the absorbance at 450 nm in each well, and the survival rate CV% of the cells in each experimental group was determined using the formula below:

$CV\%=(A_t/A_c)\times 100\%$, Where CV% stands for cell survival rate; A_t represents the absorbance value of the experimental group's cells; and A_c displays the value of the control group's inert cell population.

Supplementary Figures

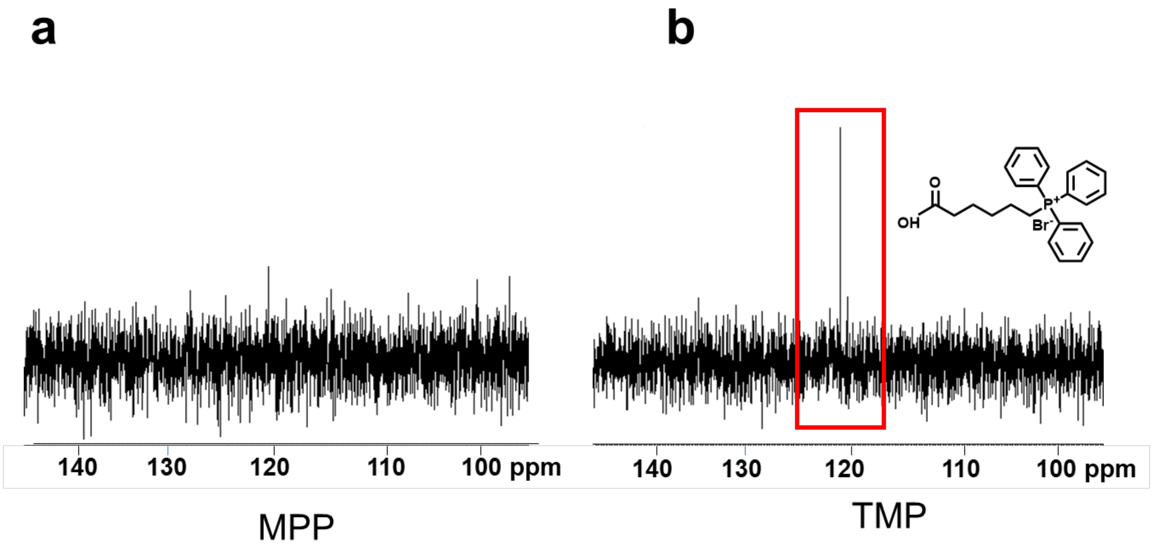

Figure. S1.
^{31}P NMR spectra of (a) MPEG-PCL-PEL (MPP) and (b) MPEG–PCL–PEI-TPP (TMP) in $CDCl_3$ solvent.
The MPP or TMP was dissolved in $CDCl_3$ with a concentration of 3 mg/mL.

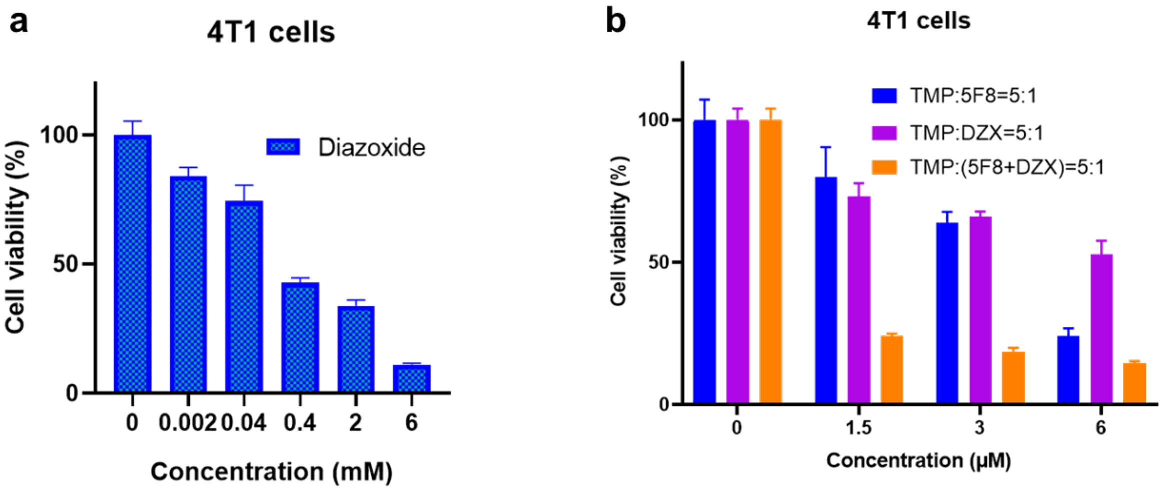

Figure. S2.
The proliferation inhibition ability of free Diazoxide (a) and different mitochondrial targeting NPs (b) in 4T1 cells.

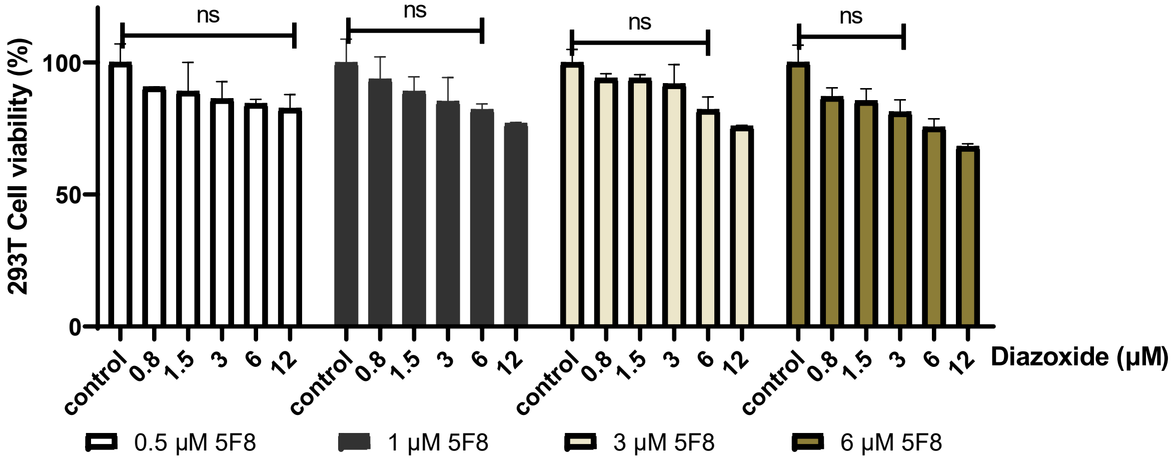

Figure. S3.

The proliferation inhibition ability after treatment with mitochondrial targeting polymer co-loaded 5F8 and Diazoxide synergism compared to mitochondrial targeting polymer loaded Diazoxide alone in 293T cells.

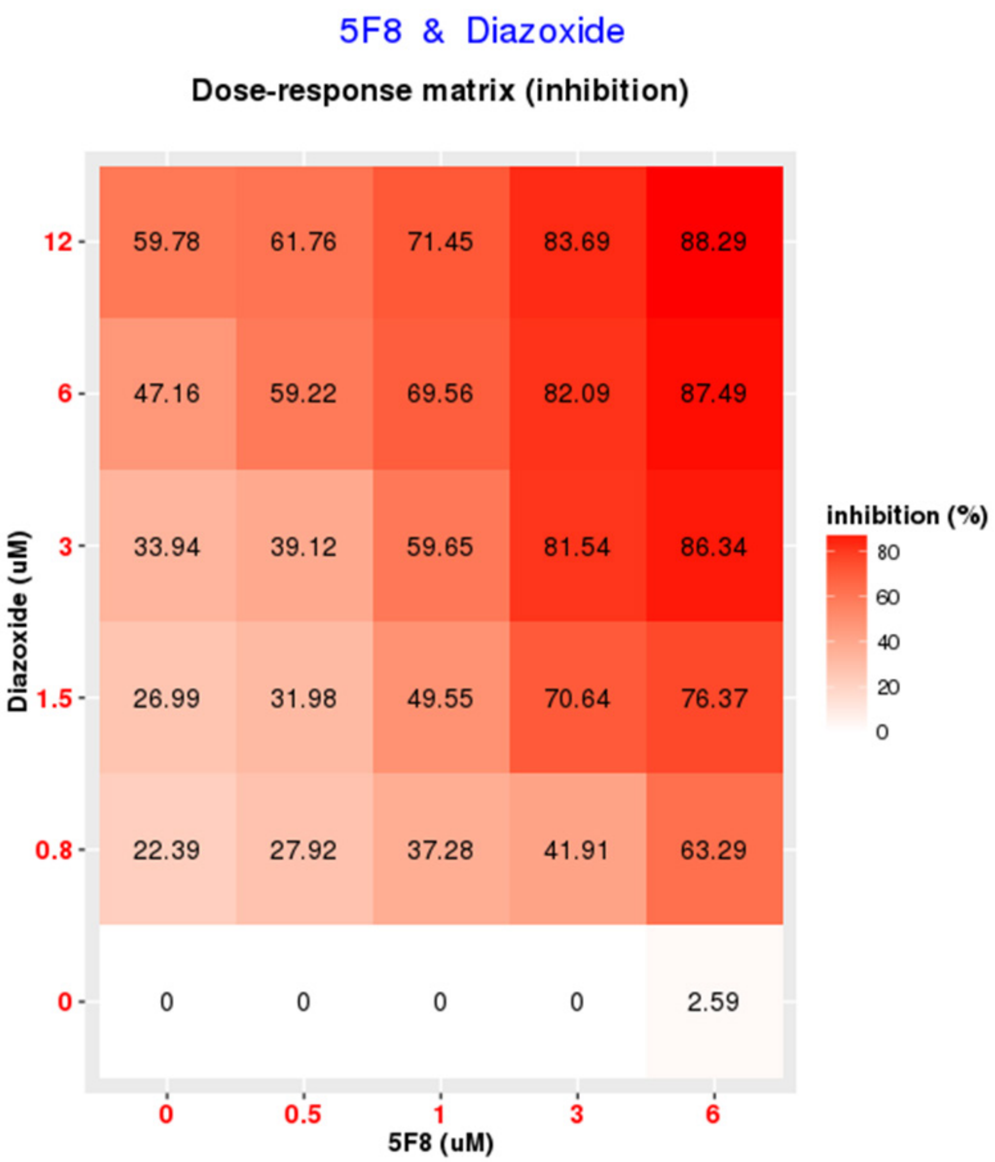

Figure. S4.
Mitochondrial targeting synergistic effect of co-loaded 5F8 and Diazoxide.

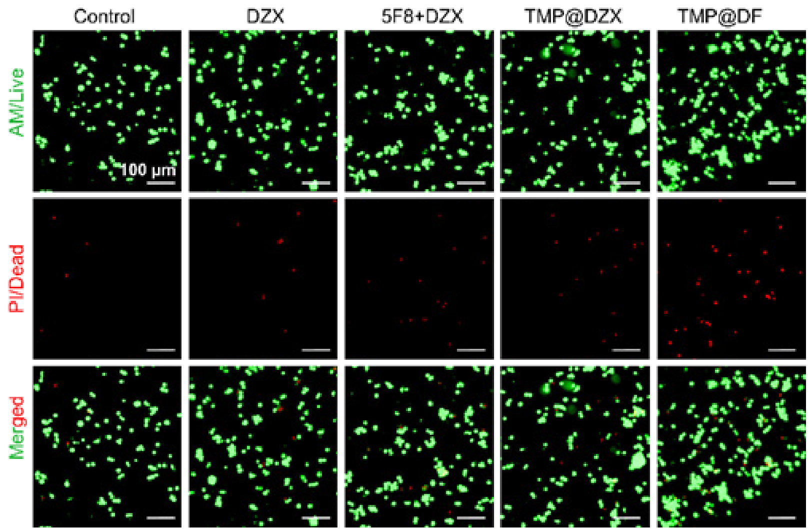

Figure. S5.
Live/dead staining fluorescence images of different treatment groups.

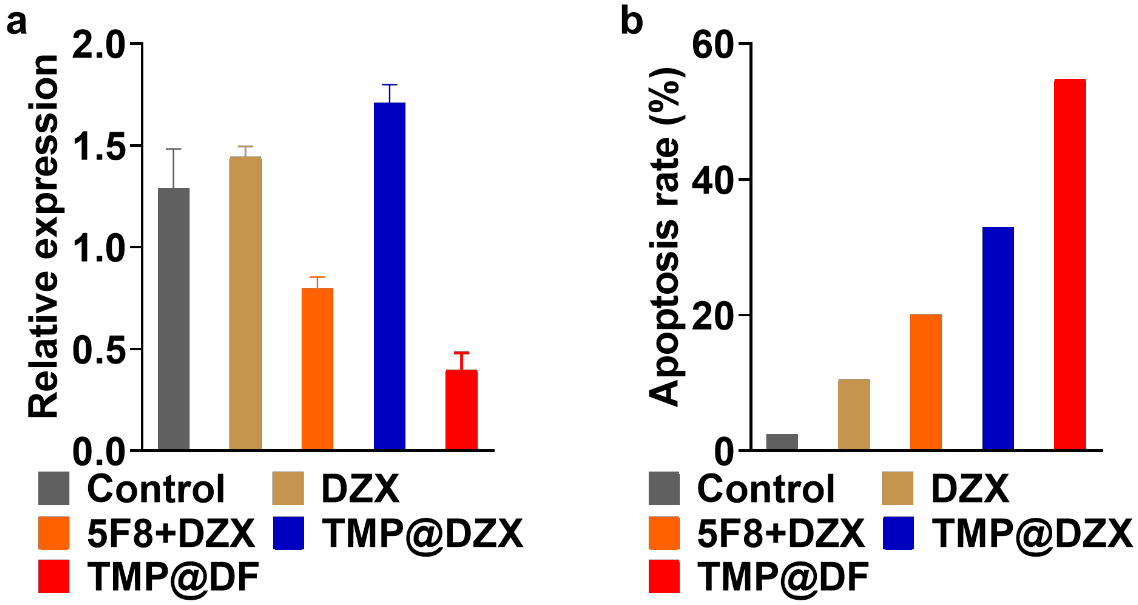

Figure. S6.
The statistical analyzed of (a) relative GPX 4 expression (Figure 4C) and (b) apoptosis rate (Figure 4I).

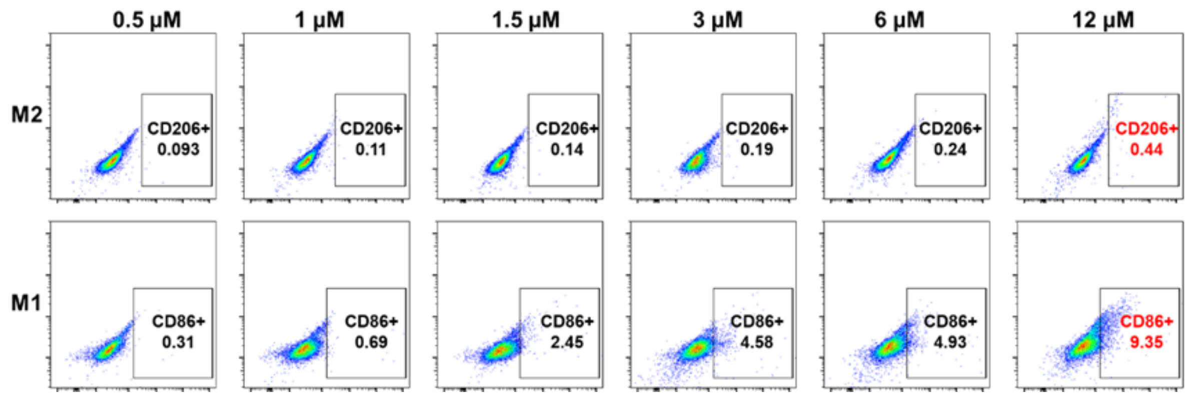

Figure. S7.
Effects of different TMP@DP NPs concentrations on macrophage phenotype.

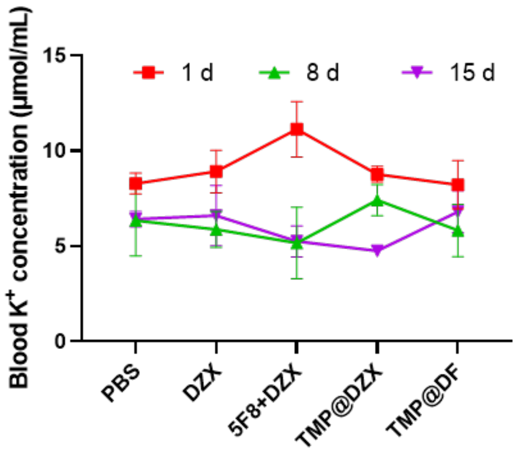

Figure S8.
The change curve of potassium concentration in serum of mice in different treatment groups at different time.
